# Biosynthetic access to the rare antiarose sugar *via* an unusual reductase-epimerase†

**DOI:** 10.1039/c9sc05766h

**Published:** 2020-03-27

**Authors:** Yijun Yan, Jing Yang, Li Wang, Dongdong Xu, Zhiyin Yu, Xiaowei Guo, Geoff P. Horsman, Shuangjun Lin, Meifeng Tao, Sheng-Xiong Huang

**Affiliations:** State Key Laboratory of Phytochemistry and Plant Resources in West China, CAS Center for Excellence in Molecular Plant Sciences, Kunming Institute of Botany, Chinese Academy of Sciences Kunming 650201 China sxhuang@mail.kib.ac.cn; State Key Laboratory of Microbial Metabolism, School of Life Sciences and Biotechnology, Shanghai Jiao Tong University 800 Dongchuan Road Shanghai 200240 China; Department of Chemistry & Biochemistry, Wilfrid Laurier University Waterloo ON N2L 3C5 Canada

## Abstract

Rubrolones, isatropolones, and rubterolones are recently isolated glycosylated tropolonids with notable biological activity. They share similar aglycone skeletons but differ in their sugar moieties, and rubterolones in particular have a rare deoxysugar antiarose of unknown biosynthetic provenance. During our previously reported biosynthetic elucidation of the tropolone ring and pyridine moiety, gene inactivation experiments revealed that RubS3 is involved in sugar moiety biosynthesis. Here we report the *in vitro* characterization of RubS3 as a bifunctional reductase/epimerase catalyzing the formation of TDP-d-antiarose by epimerization at C3 and reduction at C4 of the key intermediate TDP-4-keto-6-deoxy-d-glucose. These new findings not only explain the biosynthetic pathway of deoxysugars in rubrolone-like natural products, but also introduce RubS3 as a new family of reductase/epimerase enzymes with potential to supply the rare antiarose unit for expanding the chemical space of glycosylated natural products.

## Abstract 2

Rubrolones, isarubrolones, and rubterolones are recently isolated glycosylated tropolonids with notable biological activity.

## Graphical abstract

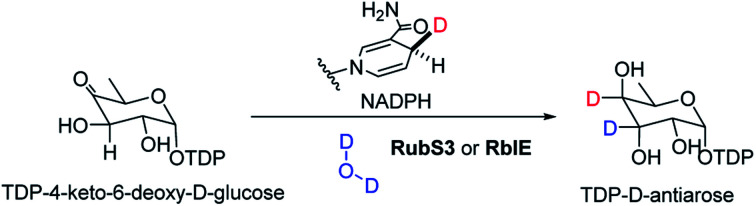

## Introduction

**Fig. 1:**
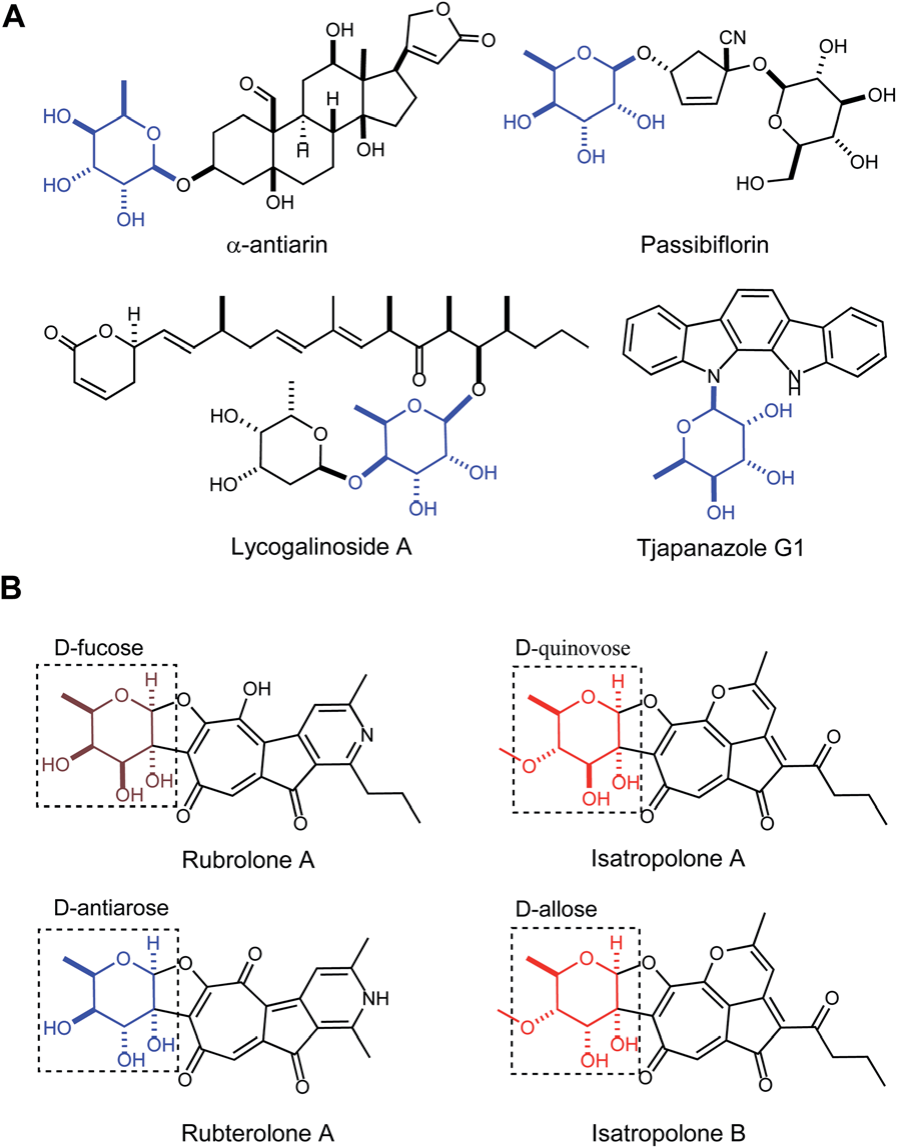
Structures of rubrolone-like compounds and selected natural products containing the d-antiarose sugar moiety. (A) Selected natural products containing d-antiarose (blue). (B) Structures of rubrolone A (contains d-fucose), isatropolone A (contains d-quinovose), isatropolone B (contains d-allose), and rubterolone A (contains d-antiarose).

Antiarose was first obtained by Kiliani from the hydrolysis of α-antiarin, a well-known poison isolated from the sap of the deadly upas tree *Antiaris toxicaria* in 1896 (Fig. 1A).^1^ After 50 years of study, the structure of this sugar was determined by Doebel along with the structure of α-antiarin in 1948.^2^ There are currently more than 50 known cardiac glycosides containing the antiarose moiety, and with nanomolar potency against Na^+^/K^+^-ATPase or human cancer cell lines, they are potentially of clinical value for treating heart failure, atrial arrhythmia and cancer.^3^ This unusual moiety is found only rarely in glycosylated natural products like passibiflorin,^4^ lycogalinoside A,^5^ and tjapanazole G1 ^6^ (Fig. 1A). However, the biosynthesis of d-antiarose remains unknown.

Tropolonoids are aromatic natural products possessing a seven-membered tropolone ring.^7^ After the tropolone alkaloid rubrolone A produced by *Streptomyces enchinoruber* was first reported in 1978 (Fig. 1B),^8^ the rubrolone family has since expanded with the identification of isatropolones^9^ and rubterolones^10^ from Actinomycete strains (Fig. 1B). All of these compounds contain a cyclopentanone ring, a tropolone unit, and a deoxysugar connected from C2 to the aglycone through a unique C–C bond. We previously demonstrated that the tropolone ring originates from type-II PKS-catalyzed chemistry and the pyridine ring is formed non-enzymatically between a 1,5-dione-containing intermediate and a free amine.^11^ To date, there are only two synthetic routes to the rubrolone aglycone^12^ and no total synthesis of any members of the rubrolone family has been reported due to the challenges of deoxysugar synthesis and attachment to the aglycone. Therefore, elucidating a biosynthetic route to deoxysugars, including sugar attachment to the aglycone, is a highly desirable goal that has remained elusive. Interestingly, rubrolones, rubterolones, and isatropolones share similar aglycone skeletons (Fig. 1B) but are decorated with different deoxysugars including d-fucose, d-antiarose (6-deoxygulose), or d-allose/d-quinovose (Fig. 1B). These closely related molecules provide an exciting opportunity to comparatively investigate and elucidate the biosynthetic origin of the rare and important antiarose moiety. Here, we report the *in vitro* characterization of RubS3–S5 and RblE in the biosynthetic pathways of rubrolone-like compounds, and establish that RubS3 and RblE are unusual reductase/epimerase catalyzing the formation of rare antiarose sugar by epimerization at C3 and reduction at C4 of the common intermediate TDP-4-keto-6-deoxy-d-glucose (**1**).

## Results and discussion

### Biochemical characterization of RubS3–S5 and identification of the products

**Fig. 2:**
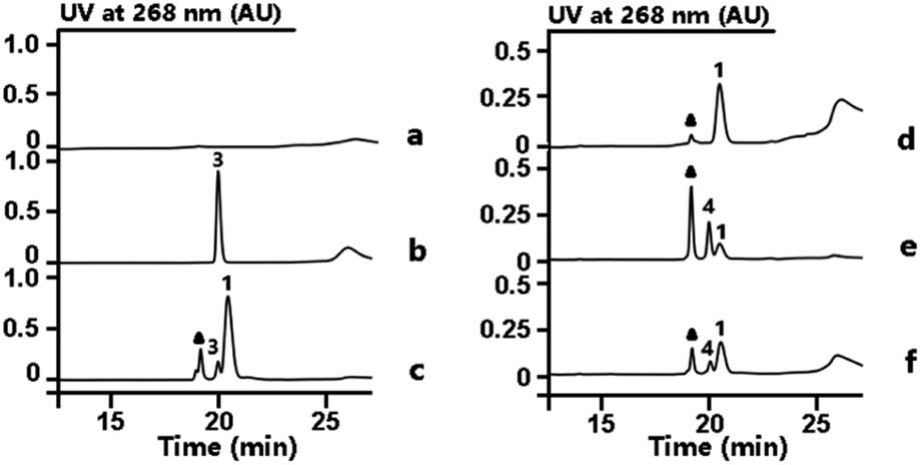
HPLC analysis of enzymatic reactions involved in TDP-sugar biosynthesis. Reactions performed as follows: (a) buffer with TTP and d-glucose-6-phosphate. (b) RubS5 reaction. (c) RubS4 reaction; (d) buffer with substrate **1** and NADPH. (e) RubS3 reaction. (f) RblE reaction. Triangle = NADP^+^.

From previous gene disruption experiments we proposed that the enzymes from RubS1 to RubS7 are involved in the biosynthesis of the rubrolone sugar moiety^11*b*^ and proposed a three-enzyme pathway to TDP-d-fucose (**2**) involving: (i) a glucose-1-phosphate thymidylyltransferase (RubS5) activing d-glucose-1-phosphate to TDP-d-glucose (**3**) (Fig. S1a†); (ii) a TDP-glucose-4,6-dehydratase (RubS4) catalyzing dehydration of **3** to form **1** (Fig. S1a†); and (iii) a NADPH dependent TDP-glucose-4-ketoreductase (RubS3) catalyzing the conversion of **1** to **2** (Fig. S1a†). To probe the biosynthetic functions of RubS3–RubS5, they were each expressed and purified (Fig. S2†). RubS5 and RubS4 were respectively identified as a thymidylyltransferase and 4,6-dehydratase by detecting **3** and **1** in the *in vitro* enzymatic reactions by HPLC and ^1^H-NMR analyses (Fig. 2, traces (b and c), Table S1†).

Incubating RubS3 with **1** and NADPH resulted in a new product **4** (Fig. 2, trace e). Subsequent large-scale preparation was carried out and the product **4** was purified for structure determination by HRESIMS and ^1^H-NMR analyses. Its HRESIMS peak at *m*/*z* 547.0723 [M–H]^−^ for C_16_H_26_N_2_O_15_P_2_ agreed with the expected mass for the reduced product **2** (calcd 547.0736). To our surprise, the ^1^H-NMR data of **4** was not identical to that of **2** reported in literature and isolated by ketoreductase Fcd reaction in our lab (Fig. S3, Table S2†).^13^ In addition, we found that the reported ^1^H NMR data for other two possible products TDP-d-allose (**5**) and TDP-d-quinovose (**6**) could not match what we observed (Table S2†),^14^ suggesting that **4** was potentially a new TDP-sugar. Further NMR analysis of **4** involved examination of the 1D and 2D spectra data (Table S3†) and proton *J* coupling constants to facilitate detailed structure elucidation. Starting from the anomeric proton at *δ*_H_ 5.54 (1H, dd, *J* = 6.9, 3.8 Hz), we assigned the remaining five proton signals of the deoxysugar moiety by the spin systems H-1/H-2/H-3/H-4 and H-5/H_3_-6 revealed by the ^1^H–^1^H COSY correlations (Fig. S4†). In contrast to the reported proton ^3^*J*-couplings of TDP-sugars **2**, **5** and **6** (Table S2†),^14^ the vicinal proton coupling constants in **4** were uniquely and uniformly small (br s or around 3.8 Hz). These small coupling constants throughout the H1–5 system of **4** indicated that all vicinal protons were oriented with dihedral angles of ∼60°, corresponding to an axial/equatorial or equatorial/equatorial configuration for each pair of vicinal protons. Similar to compounds **2**, **5** and **6**, the coupling constants for the anomeric proton (6.9, 3.8 Hz) inferred an axial orientation for TDP substitution at C-1 in **4**. Consequently, H-2 and H-5 in **4** were restricted to axial configurations, and therefore H-3 and H-4 are necessarily equatorial. Only with this interpretation can each pair of vicinal protons in **4** be absent from a dihedral angle of 180°, which would incur a large ^3^*J* constant (usually 9–11 Hz). Series of HMBC correlations of H-10/C-11, H_2_-11/C-9, H-9/C-7, H_2_-8/(C-7, C-9), H-7/(C-12, C-16), H-12/(C-15, C-16), H_3_-13/(C-12, C-14, C-15) ascertained the carbon core skeleton of the TDP subunit (Fig. S5, Table S3†). The detected ROESY correlations of H-1/H-2, H-2/H-3, H-3/H-4, H-4/H-5, and H-4/H_3_-6, in the sugar moiety of **4** required axial orientations for H-2, HO-3, and HO-4 (Fig. S5, Table S3†). Unlike the fucose sugar subunit, no ROESY cross peaks occurred between H-3 and H-5 (Fig. S5, Table S3†). Thus, the structure of the RubS3 product **4** was unambiguously assigned as TDP-d-antiarose^15^ as depicted in Fig. S5.†

### Mechanism of TDP-antiarose formation catalyzed by RubS3

**Fig. 3:**
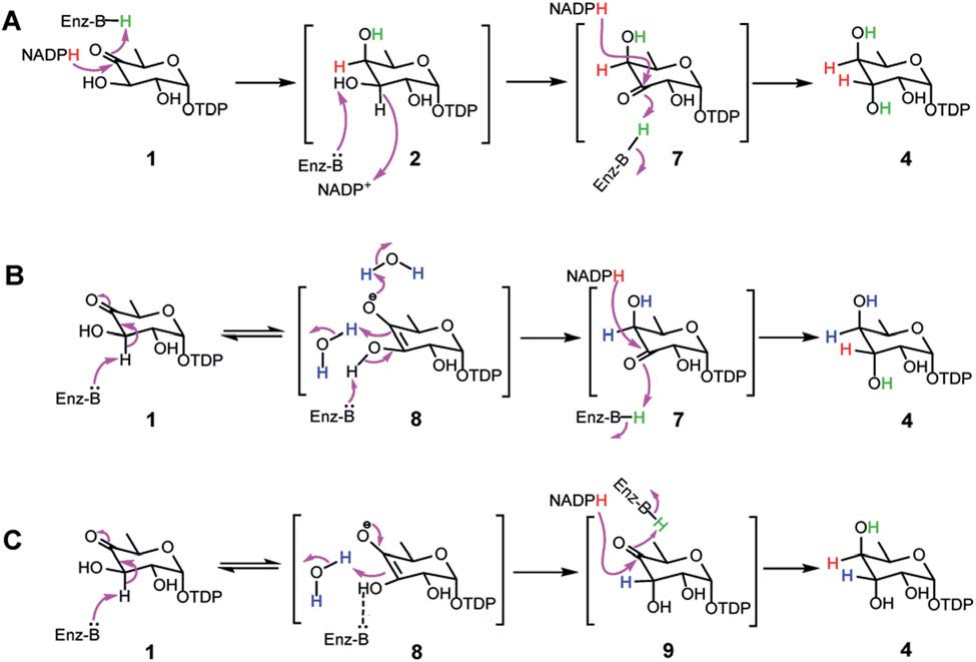
Three proposed mechanisms for formation of **4** from **1** in RubS3 mediated enzymatic reaction.

Transformation of **1** to the product **4** requires epimerization at C3 and reduction at C4, implying that RubS3 is a bifunctional TDP-deoxysugar reductase/epimerase. Sugar epimerizations often proceed through a keto intermediate; a pre-existing functional group in **1** that facilitates deprotonation/reprotonation^16^ and implies three possible mechanisms (Fig. 3). Mechanism A is initiated by NADPH attack at the *re*-face of the C4 carbonyl of **1** to afford an axial hydroxyl at C4 in the intermediate **2**. The NADP^+^ would be reduced back to NADPH by oxidation of the C3 hydroxyl to furnish a new keto functional group at C3 in intermediate **7**.^16,17^ Rotation of this intermediate in the enzyme active site would likely need to occur to expose the *si*-face to reduction by NADPH and generate an axial hydroxyl at C3 in the product **4** (Fig. 3A). Mechanism B follows the more standard epimerization logic of general base-catalyzed deprotonation at C3. The resulting enolic intermediate **8** could transform to keto intermediate **7** to produce **4** as mentioned in path A (Fig. 3B). Mechanism C would also start with general-base-catalyzed formation of the enolic intermediate **8** but instead regenerate the C4 keto group in intermediate **9** for a final reduction by NADPH to yield product **4** (Fig. 3C).

To distinguish among these three mechanisms, we first explored mechanism A by enzymatically synthesizing intermediate **2** using the ketoreductase Fcd.^13*b*^ However, no new products were observed even with high concentrations (20 μM) of RubS3 incubated with **2** and NADP^+^ (Fig. S6,† trace b), indicating that **2** is not an intermediate for the RubS3 reaction and mechanism A was ruled out. Similarly, mechanism B was discarded because no new peaks were detected when RubS3 was presented with TDP-3-keto-d-fucose (**7**) that had been synthesized using the TDP-4-keto-6-deoxy-d-glucose 3,4-ketoisomerase FdtA (Fig. S6,† trace d).^18^

After eliminating mechanisms A and B, deuterium-labeling experiments were used to evaluate mechanism C (Fig. S7†). First, the RubS3 reaction was performed in D_2_O, and the isolated product was characterized by HRMS and ^1^H-NMR analyses to characterize deuterium incorporation. HRESIMS data (Fig. S7a-I,†*m*/*z* 548.0816 [M–H]^−^, calcd for C_16_H_25_DN_2_O_15_P_2_, 548.0798) showed only one deuterium incorporated into **4**, and reduced H3 signal in the ^1^H-NMR spectrum (Fig. S7c†) indicate solvent-derived deuterium incorporation at this position. Clearly, solvent water is required for C3 epimerization, just like RmlC which contains a conserved water molecule for donation of a proton to C3,^19^ however a solvent exchangeable general acid enzyme residue in place of an actual water molecule cannot be ruled out.^20^ To map the trajectory of the hydride from NADPH, we synthesized [4*S*–^2^H]NADPH and [4*R*–^2^H]NADPH for use in the RubS3 reaction.^21^ HRMS revealed deuterium incorporation into TDP-d-antiarose only in the presence of [4*S*–^2^H]NADPH but not with [4*R*–^2^H]NADPH (Fig. S7a-II,†*m*/*z* 548.0796 [M–H]^−^, calcd for 548.0798), indicating transfer of the pro-*S* hydride from NADPH to TDP-d-antiarose. Finally, combining both [4*S*–^2^H]NADPH and D_2_O in the same RubS3-mediated *in vitro* reaction yielded HRESIMS data (Fig. S7a-III,†*m*/*z* 549.0866 [M–H]^−^, calcd for 549.0861) for the corresponding product with a molecular formula of C_16_H_24_D_2_N_2_O_15_P_2_, demonstrating incorporation of two ^2^H into **4**. Taken together, we conclude that 4*S*–H of the NADPH transfers to C4 and a solvent-derived proton ends up at C3 in TDP-d-antiarose. Moreover, NADPH-derived deuterium incorporation at H4 also rules out mechanisms A and B (Fig. 3).

### Phylogenetic and kinetic analysis of RubS3 and RblE

**Fig. 4:**
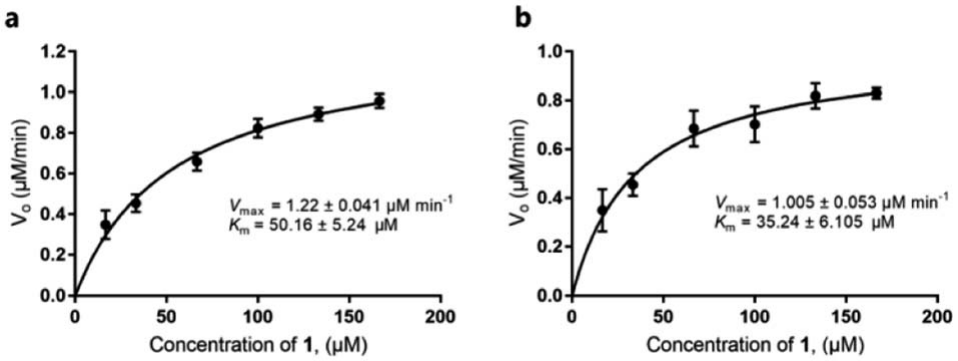
Michaelis–Menten kinetic studies of RubS3 or RblE. (a) RubS3. (b) RblE.

Our discovery of RubS3 as a novel epimerase/reductase prompted a genomic survey of RubS3 homologs. Although homologs of RubS3 with amino acid sequence identities ranging from 50% to 60% are widely distributed in bacterial genomes, RubS3 phylogenetically clusters in a clade shared by only one other enzyme – RblE (59% identity) from the rubterolone biosynthetic gene cluster (Fig. S8†).^10^ We therefore selected RblE and three other RubS3 homologues to test their functions. As expected, only RblE could convert **1** to **4** (Fig. 2, trace f) and the three other homologs were inactive (Fig. S6,† traces e–g). We then compared the catalytic efficiency between RubS3 and RblE. The spectrophotometric assay monitoring decreasing UV absorbance of NADPH was used to obtain Michaelis–Menten kinetic data for RubS3 and RblE with **1** as substrate. The RubS3-catalyzed reaction was linear over a period of 30 min when RubS3 concentrations were kept below 1.5 μM. The activity of RubS3 had a *K*_m_ of 50 μM, a *k*_cat_ of 7.7 min^−1^, and a *k*_cat_/*K*_m_ of 0.15 min^−1^ μM^−1^ (Fig. 4a), and similar kinetic parameters were obtained for RblE, with a *K*_m_ of 35 μM, a *k*_cat_ of 6.4 min^−1^, and a *k*_cat_/*K*_m_ of 0.18 min^−1^ μM^−1^ (Fig. 4b). The similar kinetic parameters strongly suggests that RblE also catalyzes the conversion of **1** to **4** en route to antiarose installation in the rubterolone biosynthetic pathway. Curiously, the rubrolone biosynthetic gene cluster also encodes RubS3 even though the strain produces rubrolones possessing d-fucose. This implicates TDP-d-antiarose as a possible intermediate in rubrolone biosynthesis, with C3 epimerization of d-antiarose by the epimerase RubS2 ^11b^ occurring after C2 keto formation (Fig. S1b†).

### Interrogation of RubS3 active site residues

We next aimed to identify active site features unique to RubS3 compared to typical TDP-sugar reductase or epimerase enzymes.^18,22^ Sequence alignments showed that RubS3 is homologous to NDP-sugar reductases rather than epimerases (Fig. S9†). Specifically, RubS3 possesses the conserved GXXGXXG sequence for NADPH binding that is characteristic of the β–α–β Rossmann-fold motif (Fig. S9†).^23^ RubS3 also has a short-chain dehydrogenases/reductases (SDR) catalytic triad composed of Thr111, Tyr135, and Lys139 (Fig. S9†).^23,24^ In this family of enzymes, Thr111 and Tyr135 bind the substrate, with Tyr135 acting as a general acid during reduction and Lys139 stabilizing the nicotinamide substrate in an active conformation. In addition, RubS3-catalyzed transfer of the pro-*S* hydride of NAD(P)H is also a general feature observed in the structures of all SDR enzymes solved to date.^25^ To test the role of the RubS3 TYK triad in reduction, three site-directed mutants were constructed (T111V, Y135F and K139A) and the activity of each mutant was compared to that of wild-type RubS3 by measuring the formation of TDP-antiarose by HPLC. The K139A substitution only moderately reduced the activity to 46.2% of the wild-type. In contrast, both T111V and Y135F substitutions resulted in markedly reduced activities. These results demonstrated a critical role for the TYK triad in RubS3 catalysis, and imply a role in C-4 ketone reduction of **1** based on known precedent for the SDR family of NDP-sugar reductases.

We next sought to identify the general base responsible for the initial step of generating the enolate intermediate. Given that His, Lys, Tyr, Ser and Glu or Asp usually fulfill this role in most epimerases,^19,20,26^ the amino acids His102, Tyr113, Arg144, Ser163 and Glu177, which are conserved in RubS3 and RblE, were selected for site-directed mutagenesis. The mutants H102V, Y113F, R144L, S163A, and E177G were constructed to probe their role in the epimerization activity in the catalysis RubS3. None of the constructed mutants abolished production of TDP-antiarose, nor did they produce any other compound, with only slightly decreased activities compared to wild-type (Fig. S10†). Our inability to identify a general base from obvious candidates suggests a potentially novel epimerization mechanism for the C-3 hydroxyl group.

## Conclusions

**Fig. 5:**
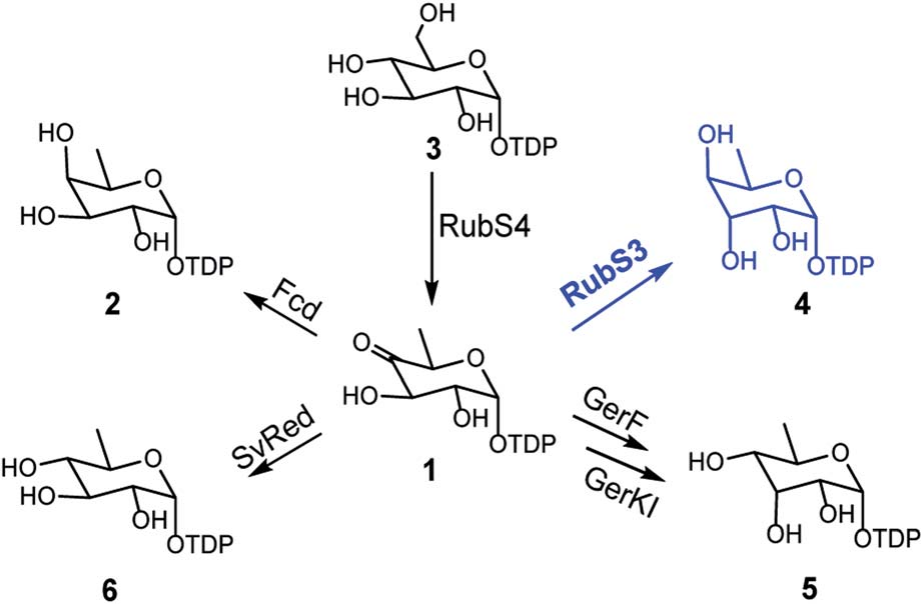
The reported biosynthetic routes for deoxysugar moieties of rubrolone-like compounds in natural products biosynthesis pathways.

The recently reported rubrolones, rubterolones and isatropolones are decorated with four different deoxysugars d-fucose, d-antiarose and d-allose/d-quinovose, respectively (Fig. 1B). Coincidently, all the four deoxysugars are the direct products of the general intermediate TDP-4-keto-6-deoxy-d-glucose (**1**) by C4-keto reduction and C3 epimerization (Fig. 5). In the literatures, TDP-d-fucose (**2**) is generated by NDP-sugar reductase Fcd-catalyzed ketoreduction of deoxysugar intermediate TDP-4-keto-6-deoxy-d-glucose (**1**);^13*b*^ TDP-d-allose (**5**) also originates from **1** by epimerase GerF and subsequent C4 ketoreduction by GerKI;^14*a*^ TDP-d-quinovose (**6**) is presumably synthesized from **1** by a *Streptomyces venezuelae* pathway-independent reductase (SvRed) (Fig. 5);^27^ TDP-antiarose can be generated inefficiently from the poor substrate TDP-fucose (Fig. S11a†)^15^ by radical *S*-adenosylmethionine enzyme DesII, which catalyzes formation of TDP-4,6-dideoxy-3-keto-d-glucose from TDP-4-amino-4,6-dideoxy-d-glucose (Fig. S11b†).^28^ In this study, we clearly demonstrated that key steps in deoxysugar biosynthesis in rubrolones and rubterolones involves the respective bifunctional epimerase/reductase enzymes RubS3 or RblE, which produce TDP-antiarose (**4**) from **1** (Fig. 5).

**Fig. 6:**
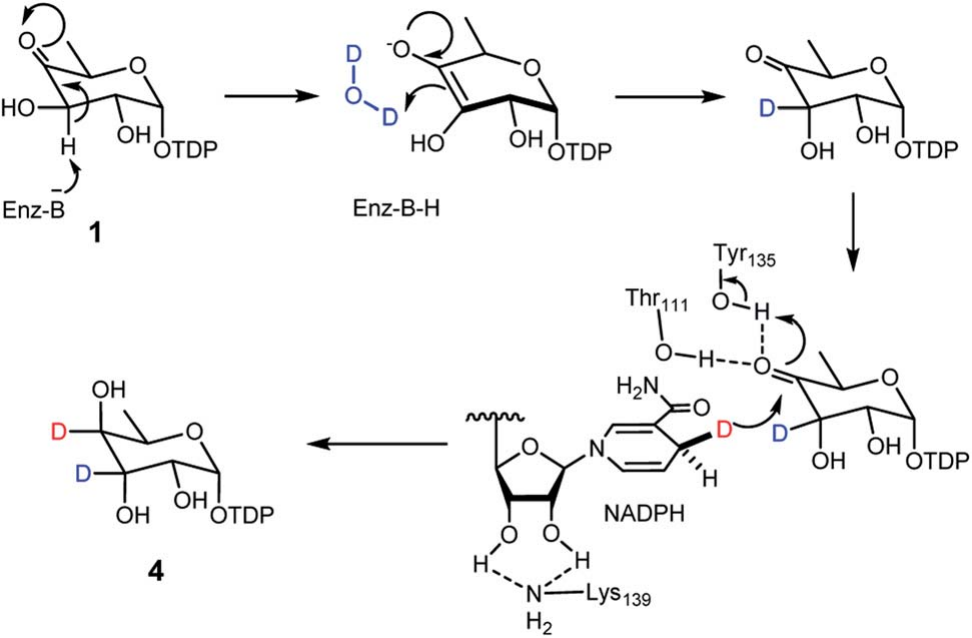
Proposed catalytic mechanism of RubS3.

The deuterium incorporation patterns and site-directed mutagenesis data suggest a simple chemical mechanism for the TDP-d-antiarose synthase reaction (Fig. 6). Initial deprotonation at C3 generates an enolate intermediate that is reprotonated by water or a solvent-exchangeable general acid on the opposite face to give the C3 epimer. Support for this mechanism includes solvent deuterium incorporation at C3 of TDP-d-antiarose, indicating that stereochemical inversion occurs *via* deprotonation/reprotonation. A final NADPH-dependent reduction of the C4 ketone to an axial hydroxyl group in the final TDP-d-antiarose product occurs analogously to the reductions catalysed by the SDR family of enzymes.^23^ We predict that Lys139 stabilizes NADPH through hydrogen bonds, and that Thr111 and Tyr135 hydroxyl groups interact with the C4 ketone of the TDP-sugar. A final reduction involves transfer of the pro-*S* hydride from NADPH to the pro-*R* face of the C4 carbonyl of the sugar nucleotide, with Tyr135 donating a proton to C4 oxygen to yield the final product TDP-d-antiarose (**4**).

Deoxysugars are the most important components of many secondary metabolites and are crucial for the antibiotic and antitumor activities of these natural products.^29^ Most naturally occurring deoxysugars are 6-deoxyhexoses, especially for bacterial glycosylated natural products.^29*b*^ To date, no more than 10 TDP-deoxysugars are directly formed from **1** by different reactions such as reduction,^13*b*,30^ isomerization,^19,20^ deoxygenation^31^ or transamination^32^ (Fig. S12†). In this study, we have expanded the palette of enzyme chemistries acting on **1** to include a new family of bifunctional enzymes catalyzing the formation of TDP-d-antiarose by C3 epimerization and reduction at C4. This study also solves the century-long puzzle of antiarose biosynthesis and will provide access to the rare antiarose unit and pave the way for engineering novel glycosylated natural products such as the cardenolide glycosides.

## Conflicts of interest

There are no conflicts to declare.

## Supplementary Material

SC-011-C9SC05766H-s001
