# Supplementary material for: Biosynthetic access to the rare antiarose sugar via an unusual reductase-epimerase
Source: Chem Sci. 2020 Mar 27;11(15):3959–64. doi: 10.1039/c9sc05766h (PMC8152690; doi:10.1039/c9sc05766h)
Supplement: SC-011-C9SC05766H-s001 [file SC-011-C9SC05766H-s001.pdf]

Electronic Supplementary Material (ESI) for Chemical Science.
This journal is © The Royal Society of Chemistry 2020

Electronic Supporting Information

Biosynthetic Access to the Rare Antiarose Sugar via an Unusual Reductase-Epimerase

Yijun Yan, ‡a Jing Yang, ‡a Li Wang, ‡a Dongdong Xu, [a] Zhiyin Yu, [a] Xiaowei Guo, [a] Geoff P. Horsman, [c] Shuangjun Lin, [b] Meifeng Tao, [b] and Sheng-Xiong Huang*[a]

[a] State Key Laboratory of Phytochemistry and Plant Resources in West China, and CAS Center for Excellence in Molecular Plant Sciences, Kunming Institute of Botany, Chinese Academy of Sciences, Kunming 650201, China
[b] State Key Laboratory of Microbial Metabolism, School of Life Sciences and Biotechnology, Shanghai Jiao Tong University, 800 Dongchuan Road, Shanghai 200240, China
[c] Department of Chemistry & Biochemistry, Wilfrid Laurier University, Waterloo, ON N2L 3C5, Canada
‡ These authors contributed equally to this work
*Correspondence and requests for materials should be addressed to S.-X.H. (email: sxhuang@mail.kib.ac.cn).

Table of Contents

Experimental procedures

Materials and general experimental procedures

Protein expression and purification

In vitro enzymatic assay of RubS3-S5 and RubS3 mutants.

Synthesis of TDP-D-fucose (**2**)

Synthesis of compound TDP-3-keto-D-fucose (**7**)

Deuterium exchange experiments

RubS3 reaction in deuterium water

RubS3 reaction using [4*S*-^{2}H]NADPH as cofactor

RubS3 reaction using [4*R*-^{2}H]NADPH as cofactor

RubS3 reaction using [4*S*-^{2}H]NADPH as cofactor in deuterium water

Kinetic analysis of RubS3 and RblE

Phylogenetic and sequence analysis

Tables

Table S1. NMR data of compounds **1-3** in D_2O

Table S2. ^{1}H-NMR data of compounds **2, 4-6** in D_2O

Table S3. NMR data of compound **4** in D_2O

Table S4. Strains, plasmids and primers used and generated in this study

Figures

Figure S1. Proposed pathways for the formation of deoxysugar dTDP-2-keto-D-fucose in rubrolone biosynthesis

Figure S2. SDS-PAGE analysis of proteins

Figure S3. Enlargerd.^{1}H-NMR spectra of compounds **2** and **4** in D_2O

Figure S4. Enlarged ^{1}H-^{1}H COSY NMR spectrum of compound **4** in D_2O

Figure S5. Key ^{1}H-^{1}H COSY, HMBC and ROESY correlations of compound **4**.

Figure S6. HPLC analysis of the products by enzymes

Figure S7. Deuterium exchange experiments

Figure S8. Phylogenetic analysis of RubS3 and its selected homologues

Figure S9. Amino acid sequences alignment

Figure S10. In vitro enzyme assays of mutants

Figure S11. Radical *S*-adenosylmethionine dependent enzyme DesII-catalyzed reactions

Figure S12. TDP-deoxysugars directly formed from TDP-4-keto-6-deoxy-D-glucose (**1)** by different reactions

Figure S13. HRESIMS analysis of compound **3**

Figure S14. HRESIMS analysis of compound **1**

Figure S15. HRESIMS analysis of compound **4**

Figure S16. HRESIMS analysis of compound **2**

Figure S17. HRESIMS analysis of compound **7**

Figure S18. HRESIMS analysis of compound **4’** formed with RubS3 in D_2O

Figure S19. HRESIMS analysis of synthesized [4*S*-^{2}H] NADPH

Figure S20. HRESIMS analysis of **4’’** formed with RubS3 use [4*S*-^{2}H] NADPH as cofactor

Figure S21. HRESIMS analysis of synthesized [4*R*-^{2}H] NADPH

Figure S22. HRESIMS analysis of compound **4** formed with RubS3 use [4*R*-^{2}H] NADPH as cofactor

Figure S23. HRESIMS analysis of compound **4’’’** formed with RubS3 in D_2O and use [4*S* ^{2}H]NADPH as cofactor

Figure S24. ^{1}H NMR spectrum of compound **3** in D_2O

Figure S25. ^{1}H NMR spectrum of compound **1** in D_2O

Figure S26. ^{1}H-^{1}H COSY NMR spectrum of compound **1** in D_2O

Figure S27. ^{1}H NMR spectrum of compound **4** in D_2O

Figure S28. ^{13}C NMR spectrum of compound **4** in D_2O

Figure S29. ^{1}H-^{1}H COSY NMR spectrum of compound **4** in D_2O

Figure S30. HSQC NMR spectrum of compound **4** in D_2O

Figure S31. HMBC NMR spectrum of compound **4** in D_2O

Figure S32. ROESY NMR spectrum of compound **4** in D_2O

Figure S33. ^{31}P NMR spectrum of compound **4** in D_2O

Figure S34. ^{1}H NMR spectrum of compound **2** in D_2O

Figure S35. ^{1}H NMR spectrum of compound **4’** in D_2O

References

Experimental procedures

Materials and general experimental procedures

The reagents, solvents, and restriction enzymes were purchased from standard commercial sources and used directly. The TDP-4-keto-6-deoxy-D-glucose was purchased from Carbosynth China Ltd. DNA isolation and manipulation in *Streptomyces* were performed according to standard protocols.[1] PCR amplifications were carried out on Biometra professional thermocycler (070-851, An Analytik Jena Company) using either Taq DNA polymerase (TaKaRa) or Pfu DNA polymerase (Vazyme). Glucose-6-phosphate dehydrogenase (Coolaber) and active recombinant bacterial alcohol dehydrogenase (Biovision) were purchased from Beijing Lablead Biotechnology Co. Ltd. Primer synthesis and DNA sequencing were performed at TsingKe Company.

HPLC analysis was conducted on a HITACHI Chromaster system equipped with a DAD detector, a Dionex carbopac PA10 carbohydrate column (4 x 250 mm, Thermo), and a flow rate of 1.0 mL/min at a column temperature of 28°C. NMR spectra were recorded in D_2O using a Bruker Ascend 800 spectrometer (Bruker Corp.), and TMS was used as internal standard. HRESIMS data were obtained using an Agilent G6230 Q-TOF mass instrument (Agilent Corp.).

Protein expression and purification

The genes encoding RubS3, RubS4 and RubS5 were amplified by PCR from genomic DNA of *Streptomyces* sp. KIB-H033 with primers listed in Table S3. The genes encoding dTDP-4-keto-6-deoxy-D-glucose reductase Fcd, dTDP-4-keto-6-deoxy-D-glucose 3,4-ketoisomerase FdtA, RblE, three homologs (SFQ20469, WP009948706, and WP094006909) and nine RubS3 mutants (H102V, T111V, Y113F, Y135F, K139A, R144L, W160T, S163A and E177G) were synthesized by GENEWIZ company. The genes were cloned into the pET-26b vector using the NdeI and XhoI (HindIII) restriction sites. The resulting constructs were used to transform *Escherichia coli* BL21(DE3) cells, and cultivated in 500 mL LB media containing kanamycin (50 μg/mL) for 4 h at 37 °C until the OD_{600} reached 0.6. The cultures were cooled to 16 °C and induced with 0.25 mM isopropyl-*β*-D-thiogalactopyranoside (IPTG) for 18 h at 16 °C. The cells were centrifuged for 10 min at 4,000 rpm at 4 °C and the pellet resuspended in 50 mL of lysis buffer (50 mM Tris, 300 mM NaCl, 15 mM imidiazole, 10% glycerol, pH 8.0) and lysed on ice by sonication. The cell lysates were centrifugated at 24,000 rpm for 30 min and the supernatant was filtered and purified using the AKTA pure system with a 5 mL Histrap™ FF column (GE Healthcare). The target proteins were desalted using a PD-10

desalting column (GE Healthcare) and concentrated by ultrafiltration using Amicon Ultra-4 (10 K, Millipore) and stored at -80 °C in buffer (100 mM NaH_2PO_4,10% glycerol, pH 7.2). Protein concentrations were determined using the Bradford method. SDS-PAGE analysis of proteins are shown in Fig S2.

***In vitro* enzymatic assay of RubS3-S5 and RubS3 mutants.**

The RubS5-catalyzed reaction was carried out in a 200 μL reaction mixture containing 50 mM Tris/HCl (pH 7.5), 1.25 mM D-glucose-1-phosphate, 2 mM dTTP, 10 μM RubS5. The RubS4-catalyzed reaction mixture (200μL) contained 50 mM Tris/HCl (pH 7.5), 10 mM $MgCl_2$, 1.25 mM D-glucose-1-phosphate, 2 mM dTTP, 2 mM NADPH, 10 μM RubS5 and 10 μM RubS4. The RubS3 and mutants reactions were performed in 200 μL system including 50 mM Tris/HCl (pH 7.5), 1 mM TDP-4-keto-6-deoxy-D-glucose (**1**), 2 mM NADPH, and 10 μM RubS3 or mutants. After incubation at 30 °C for 2 h, the reactions were quenched by adding 50 μL chloroform. The reaction mixtures were then centrifuged at 12,000 rpm for 5 min and the supernatants were analyzed by analytical HPLC. The HPLC analysis was performed at a flow rate of 1 mL/min with UV detection at 278 nm using a 28 min solvent gradient as follows: 5% B (0-5 min); 20% B (5-10 min); 40% B (10-15 min); 60% B (15-20 min); 80% B (20-22 min); 100% B (22-25 min); 5% B (25-28 min). {A: H_2O; B: acetic acid – ammonium acetate buffer (700 mM, pH 5.2)}.

The RubS3-, RubS4- and RubS5-catalyzed reactions were each scaled up to a 6 mL volume. After incubation at 30 °C for 2 h, the enzymatic reactions were quenched by chloroform and centrifuged, and the supernatant was evaporated and the compounds were isolated by analytical HPLC using a Dionex carbopac PA10 carbohydrate column. The structures of **1**, **3** and **4** were determined by analyses of HRESIMS data (Figs. S13-S15) and NMR spectra (Figs. S24-S33).

Compound **1** (0.9 mg), produced by RubS4, white powder; ^{1}H-NMR data see Table S1; HRESIMS *m/z* 545.0588 $[M-H]^-$ for $C_{16}H_{24}N_2O_{15}P_2$ (calcd. 545.0579).

Compound **3** (1.0 mg), produced by RubS5, white powder; ^{1}H-NMR data see Table S1; HRESIMS *m/z* 563.0682 $[M-H]^-$ for $C_{16}H_{26}N_2O_{16}P_2$ (calcd. 563.0685).

Compound **4** (1.2 mg), produced by RubS3, white powder; 1D and 2D NMR data see Table S3; HRESIMS *m/z* 547.0723 $[M-H]^-$ for $C_{16}H_{26}N_2O_{15}P_2$ (calcd. 547.0736).

Synthesis of TDP-D-fucose (2)

The TDP-D-fucose (**2**) was synthesized by TDP-4-keto-6-deoxy-D-glucose reductase Fcd, and the reaction was carried out in a 6 mL reaction mixture containing 50 mM Tris/HCl (pH

7.5), 1mM TDP-4-keto-6-deoxy-D-glucose (**1**), 2 mM NADPH, and 10 μM Fcd. The isolation was performed as above for RubS3-catalyzed reactions. The structure of **2** was determined by analyses of HRESIMS data (Fig S16) and ^{1}H-NMR spectrum (Fig. S34).

Compound **2** (0.8 mg) produced by Fcd; ^{1}H-NMR data see Table S1; HRESIMS *m/z* 547.0754 $[M-H]^-$ for $C_{16}H_{26}N_2O_{15}P_2$ (calcd. 547.0736).

Synthesis of compound TDP-3-keto-D-fucose (7)

The TDP-3-keto-D-fucose (**7**) was obtained from the FdtA-catalyzed reaction using **1** as the substrate in a 1 mL mixture. Compound **7** was purified using the method mentioned above for compound **3**. The structure of **7** was determined by analysing HRESIMS data (Fig. S17).

Compound **7** (0.15 mg) produced by FdtA; HRESIMS *m/z* 545.0587 $[M-H]^-$ for $C_{16}H_{24}N_2O_{15}P_2$ (calcd. 545.0579).

Deuterium exchange experiments.

RubS3 reaction in deuterium water: The reaction mixture of RubS3 contained 750 μL 0.4 M Tris/HCl (pH 7.5, final 50 mM), 150 μL 40 mM TDP-4-keto-6-deoxy-D-glucose (**1**) (final 1 mM), 300 μL 40 mM NADPH (final 2mM), 60 μL 1 mM RubS3 (final 10 μM), and 4740 μL deuterium water. After incubation at 30 °C for 2 h, the enzymatic reaction was quenched by chloroform and centrifuged, the supernatant was evaporated and the compound was isolated by analytical HPLC using a Dionex carbopac PA10 carbohydrate column. The structure of **4'** was determined by analysing ^{1}H-NMR (Fig. S35) and HRESIMS data (Fig. S18).

Compound **4'** (1.1 mg), white powder; ^{1}H-NMR data see Fig. S35; HRESIMS *m/z* 548.0816 $[M-H]^-$ for $C_{16}H_{25}DN_2O_{15}P_2$ (calcd. 548.0798).

RubS3 reaction using [4*S*-^{2}H] NADPH as cofactor: [4*S*-^{2}H] NADPH was synthesized using the method reported by Barber.[2] The reaction (200 μL) contain 83 mM phosphate buffer (pH 8.0), 9.3 mM $NADP^+$, 14.7 mM D-glucose-1-^{2}H, 40% DMSO and 5 units of glucose-6-phosphate dehydrogenase. After incubation at 30 °C for 1 h, 5 μL reaction solution was used for HRESIMS analysis (Fig. S19, HRESIMS *m/z* 745.0924 $[M-H]^-$ for $C_{21}H_{29}DN_7O_{17}P_3$, calcd for 745.0901). Then, other 20 μL reaction solution was added to the RubS3 reaction system including 50 mM Tris/HCl (pH 7.5), 1 mM TDP-4-keto-6-deoxy-D-glucose (**1**), and 10 μM RubS3. The enzymatic reaction was quenched by chloroform and centrifuged, the supernatant was isolated by analytical HPLC using a Dionex carbopac PA10 carbohydrate column. The structure of **4"** was determined by analysing HRESIMS data (Fig. S20).

Compound **4"**, HRESIMS *m/z* 548.0796 $[M-H]^-$ for $C_{16}H_{25}DN_2O_{15}P_2$ (calcd. 548.0798).

RubS3 reaction using [4*R*-^{2}H]NADPH as cofactor: [4*R*-^{2}H] NADPH was synthesized by the

method of Barber.[2] The reaction (200 μL) contained 25 mM Tris buffer (pH 9.0), 2.8 mM $NADP^+$, 1 M 2-propanol-$^2H_8$, and 5 units of alcohol dehydrogenase. After incubation at 30 °C for 1 h, 5 μL reaction solution was used for HRESIMS analysis (Fig. S21, HRESIMS *m/z* 372.0436 $[M-2H]^{2-}$ for $C_{21}H_{29}DN_7O_{17}P_3$, calcd for 372.0414) and other 20 μL reaction solution was added to the RubS3 reaction system containing 50 mM Tris/HCl (pH 7.5), 1 mM TDP-4-keto-6-deoxy-D-glucose, and 10 μM RubS3. This reaction was quenched by chloroform and centrifuged, the supernatant was isolated by analytical HPLC using a Dionex carbopac PA10 carbohydrate column. The structure of **4** was determined by analysing HRESIMS data (Fig. S22).

Compound **4,** HRESIMS *m/z* 547.0700 $[M-H]^-$ for $C_{16}H_{26}N_2O_{15}P_2$ (calcd. 547.0736).

RubS3 reaction using [4*S*-^{2}H]NADPH as cofactor in deuterium water: The 200 μL reaction mixture of RubS3 was prepared by adding 20 μL glucose-6-phosphate dehydrogenase reaction solution, 25 μL 0.4 M Tris/HCl (pH 7.5), 5 μL 40 mM TDP-4-keto-6-deoxy-D-glucose (**1**), 2 μL 1 mM RubS3, and 148 μL deuterium water. After incubation at 30 °C for 2 h, the enzymatic reaction was quenched by chloroform and centrifuged, the supernatant was isolated by analytical HPLC using a Dionex carbopac PA10 carbohydrate column. The structure of **4'''** was determined by analysing HRESIMS data (Fig. S23).

Compound **4'''**, 549.0866 $[M-H]^-$ for $C_{16}H_{24}D_2N_2O_{15}P_2$ (calcd. 549.0861).

Kinetic analysis of RubS3 and RblE

The RubS3 or RblE concentration was 0.005 mg/mL (0.16 μM) in the reaction mixture contained 50 mM Tris/HCl (pH 7.5), 0.5 mM NADPH and TDP-4-keto-6-deoxy-D-glucose (**1**) concentrations ranging between 20 μM and 200 μM. The kinetics was measured by the decrease in absorbance at 340 nm using an extinction coefficient of 6220 $M^{-1}cm^{-1}$ for NADPH. Recordings were carried out with a NanoDrop instrument (Fisher Scientific). All assays were performed in triplicate, the K_M and V_{max} values were calculated from curve fitting to the Michaelis-Menten equation $v_0 = (V_{max} \times [S])/(K_M + [S])$. The k_{cat} values were calculated according to the equation $k_{cat} = V_{max}/[E]$.

Phylogenetic and sequence analysis

The sequence data was analyzed by the neighbor-joining method using the NEIGHBOR program Phylogeny Inference Package (PHYLIP).[3] Bootstrapping and decay analysis were performed by NJ plot. Parsimony analysis and various clades were determined by MEGA.[4] The sequence alignment was created using Clustal Omega[5] and the figure was produced using EsPript 3.0.[6]

Table S1. NMR data of compounds **1-3** in D_2O. (δ in ppm, J in Hz).

No.	**1**	**2**	**3**
	δ_H	δ_H	δ_H
1	5.55 (dd, 6.8, 3.6)	5.54 (m)	5.54 (d, 2.3)
2	3.62 (d, 9.8)	3.73 (dt, 10.4, 3.2)	3.47 (d, 9.6)
3	3.78 (d, 9.8)	3.89 (dd, 10.4, 3.0)	3.40 (t, 9.6)
4		3.80 (br s)	3.72 (d, 10.1)
5	4.10 (q, 6.5)	4.25 (br q, 6.5)	3.84 (d, 10.1)
6	1.22 (d, 6.5)	1.19 (d, 6.5)	3.71 (d, 12.8)<br>3.80 (d, 12.8)
7	6.35 (t, 6.8)	6.32 (t, 6.9)	6.29(t, 6.9)
8	2.35 (m)	2.36 (m)	2.32 (m)
9	4.63 (m)	4.59 (m)	4.56 (m)
10	4.18-4.19 (overlapped)	4.14-4.16 (overlapped)	4.12-4.13 (overlapped)
11	4.18-4.19 (overlapped)	4.14-4.16 (overlapped)	4.12-4.13 (overlapped)
12	7.75 (s)	7.71 (s)	7.68 (s)
13	1.93 (s)	1.90 (s)	1.87 (s)

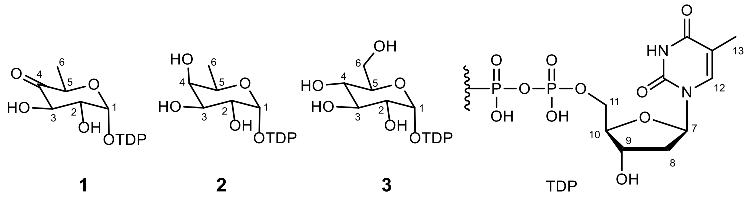

Table S2. NMR data of compound **4** in this study and the NMR data of compounds **2**, **5**, and **6** reported in the literatures[a]

No.	TDP-D-antiarose (**4**)	TDP-D-fucose (**2**)[7]	TDP-D-allose (**5**)[8]	TDP-D-quinovose (**6**)[7]
	δ_H	δ_H	δ_H	δ_H
1	5.54 (dd, 6.9, 3.8)	5.56 (dd, 6.8, 3.6)	5.38 (dd) J_p 7.15; $J_{1,2}$ 3.75	5.53 (dd, 7.0, 3.6)
2	3.90 (br s)	3.74 (dt, 10.5, 3.2)	3.65 (dd) $J_{2,3}$ 3.6	3.52 (dt, 9.5, 3.6)
3	3.96 (t, 3.8)	3.91 (dd, 10.5, 3.2)	3.95 (m) $J_{3,4}$ 3.2	3.71 (t, 9.5)
4	3.74 (d, 3.8)	3.81 (br d, 3.2)	3.25 (dd) $J_{4,5}$ 10.1	3.15 (t, 9.5)
5	4.45 (q, 6.6)	4.28 (br q, 6.6)	4.02 (m) $J_{5,6}$ 6.3	3.97 (dq, 9.5, 6.2)
6	1.21 (d, 6.6)	1.21 (d, 6.6)	1.13 (d)	1.27 (d, 6.2)
7	6.34 (t, 7.0)	6.34 (t, 7.0)	6.24 (t)	6.34 (t, 6.9)
8	2.37 (m)	2.36 (m)	2.24 (m)	2.36 (m)
9	4.62 (m)	4.62 (m)	4.48 (m)	4.62 (m)
10	4.17-4.19[b]	4.17 (m)	4.06 (m)	4.17 (m)
11	4.17-4.19[b]	4.17 (m)	4.04 (dd)	4.17 (m)
12	7.74 (s)	7.74 (s)	7.59 (s)	7.74 (s)
13	1.93 (s)	1.93 (s)	1.78 (s)	1.93 (s)

[a] Measured in D_2O, δ in ppm, J in Hz. [b] Overlapped signals.

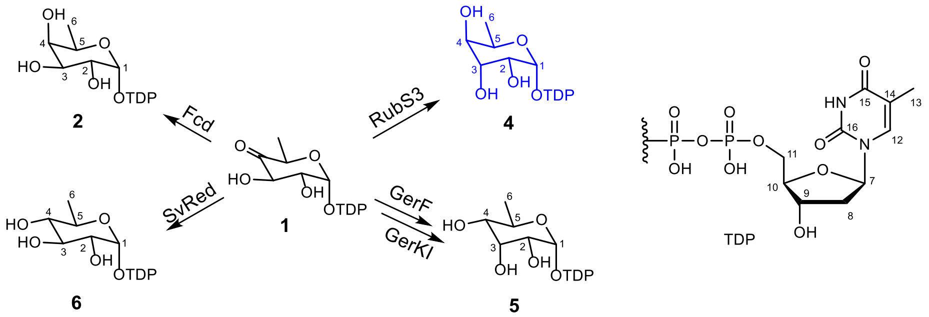

Table S3. NMR data of compound **4** in D_2O. (δ in ppm, J in Hz).

No.	TDP-D-antiarose(**4**)				
	δ_C	δ_H	^1H-^1H COSY	HMBC	ROESY
1	95.39	5.54 (dd, 6.9, 3.8)	2	3, 5	2
2	64.56	3.90 (br s)	1, 3		1, 3
3	70.35	3.96 (t, 3.8)	2, 4		4
4	71.48	3.74 (d, 3.8	3	2, 3	3, 5, 6
5	63.29	4.45 (q, 6.6)	6	1, 4, 6	4, 6
6	14.82	1.21 (d, 6.6)	5	4, 5	4, 5
7	84.95	6.34 (t, 7.0)	8	12, 16	8, 10
8	38.51	2.37 (m)	7, 9	7, 9	7, 9, 12
9	70.92	4.62 (m)	8, 10	7	
10	65.43	4.17-4.19[a]	9	11	
11	85.31	4.17-4.19[a]		9	
12	137.35	7.74 (s)		7, 13, 15, 16	8, 13
13	11.62	1.93 (s)		12, 14, 15	12
14	111.75				
15	166.58				
16	151.73				

[a] Overlapped signals.

Table S4. Strains, plasmids and primers used and generated in this study

Strains/Plasmid	Purpose	Sources
Strains		
E. coli		
DH10B	Host strain for cloning	Invitrogen
BL21(DE3)	Heterologous host for protein expression	NEB
Streptomyces		
Streptomyces sp. KIB-H033	Rubrolones wild type producing strain	This study
Plasmids		
pET26b(+)	Kan^r , Protein expression vector used in *E.coli*, encoding C-terminal His-tag,	Novagen
pET26b-rubS5	pET26b(+) derived plasmid for expression C-terminal His-tag RubS5	This study
pET26b-rubS4	pET26b(+) derived plasmid for expression C-terminal His-tag RubS4	This study
pET26b-rubS3	pET26b(+) derived plasmid for expression C-terminal His-tag RubS3	This study
pET26b-rblE	pET26b(+) derived plasmid for expression C-terminal His-tag RblE	This study
pET26b-fcd	pET26b(+) derived plasmid for expression C-terminal His-tag Fcd	This study
pET26b-fdtA	pET26b(+) derived plasmid for expression C-terminal His-tag FdtA	This study
pET26b-sfq20469	pET26b(+) derived plasmid for expression C-terminal His-tag SFQ20469	This study
pET26b-w8706	pET26b(+) derived plasmid for expression C-terminal His-tag WP009948706	This study
pET26b-w6909	pET26b(+) derived plasmid for expression C-terminal His-tag wp094006909	This study
Primers		
RubS5-pET26b-S	5'- GGAATTCCATATGAAAGGGATCATCCTCG-3'	This study
RubS5-pET26b-A	5'- CCGCTCGAGGCCGGCCTGCGCCGC -3'	This study
RubS4-pET26b-S	5'- GGAATTCCATATGTCGCGACAGCTGCGGATCCTG-3'	This study
RubS4-pET26b-A	5'- CCGCTCGAGTGCCCCCCGGGCCGGGGCG -3'	This study
RubS3-pET26b-S	5'- GGAATTCCATATGATGAGGAAGGCGATCTG-3'	This study
RubS3-pET26b-A	5'- CCGCTCGAGGGCCCGCTGGACGGC-3'	This study

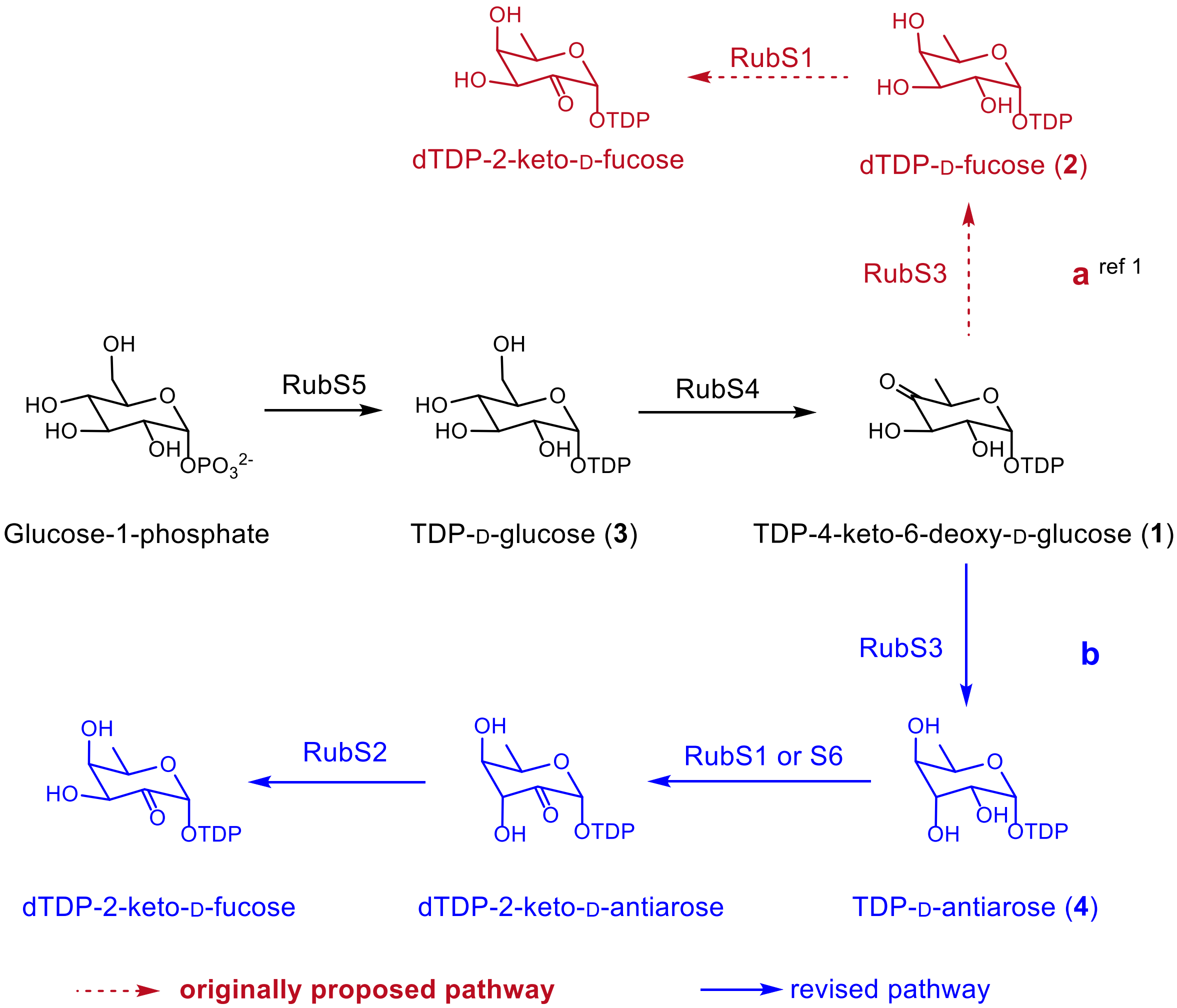

Figure S1. Proposed pathways for the formation of deoxysugar dTDP-2-keto-D-fucose in rubrolone biosynthesis. **a** originally proposed. **b** revised in this study.

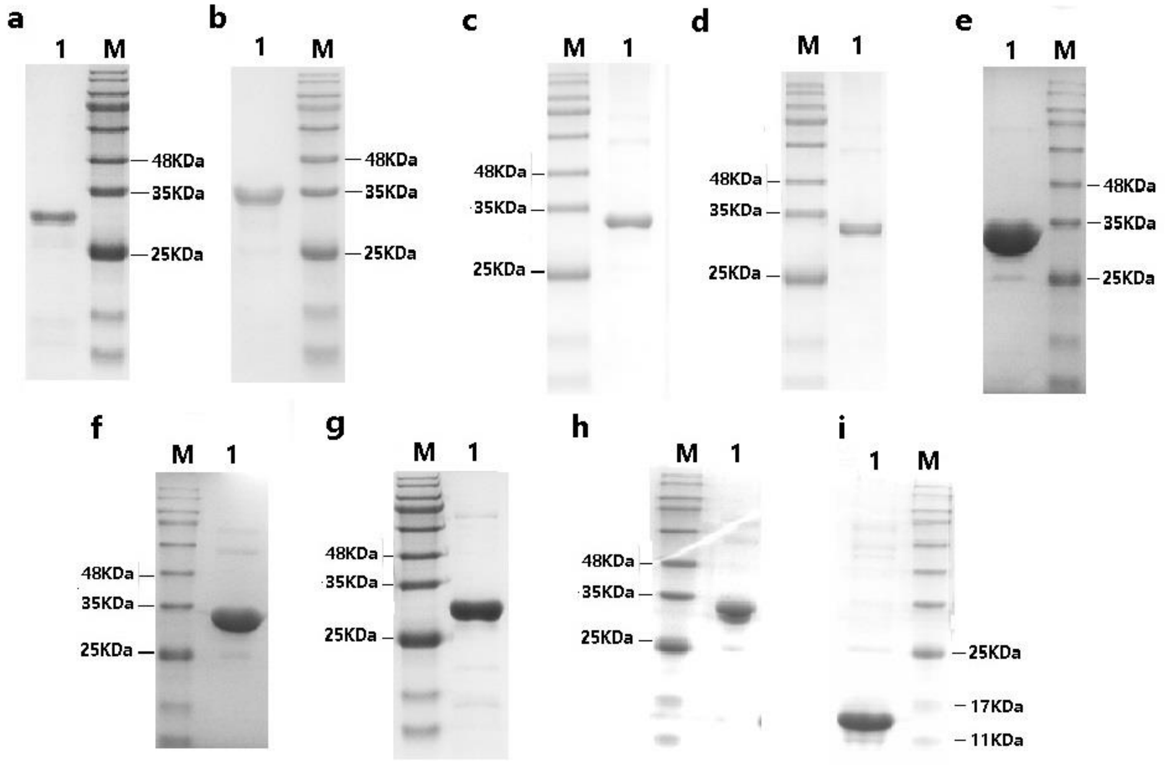

Figure S2. SDS-PAGE analysis of proteins. **a**. Lane 1, RubS5 (calculated molecular weight 31.5 KDa); **b**. Lane 1, RubS4 (calculated molecular weight 37.4 KDa); **c**. Lane 1, RubS3 (calculated molecular weight 32.9 KDa). **d**. Lane 1, RblE (calculated molecular weight 32.9 KDa). **e**. Lane 1, SFQ20469 (calculated molecular weight 32.6 KDa). **f**. Lane 1, WP009948706 (calculated molecular weight 32.5 KDa). **g**. Lane 1, WP094006909 (calculated molecular weight 31.5 KDa). **h**. Lane 1, Fcd (calculated molecular weight 35.5 KDa). **i**. Lane 1, FdtA (calculated molecular weight 16.0 KDa).

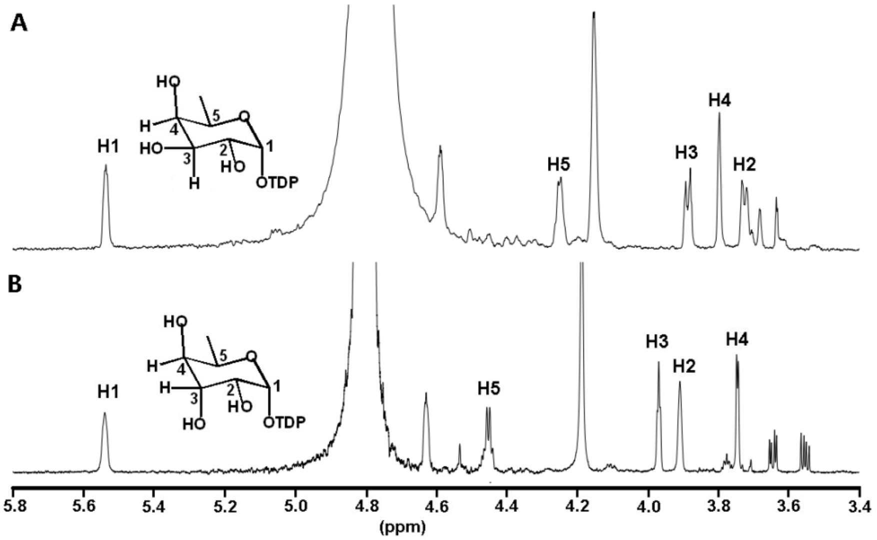

Figure S3. Enlargerd^{1}H-NMR spectra of compounds **2** and **4** in D_2O. **A** ^{1}H-NMR of TDP-D-Fucose (**2**). **B** ^{1}H-NMR of TDP-D-antiarose (**4**).

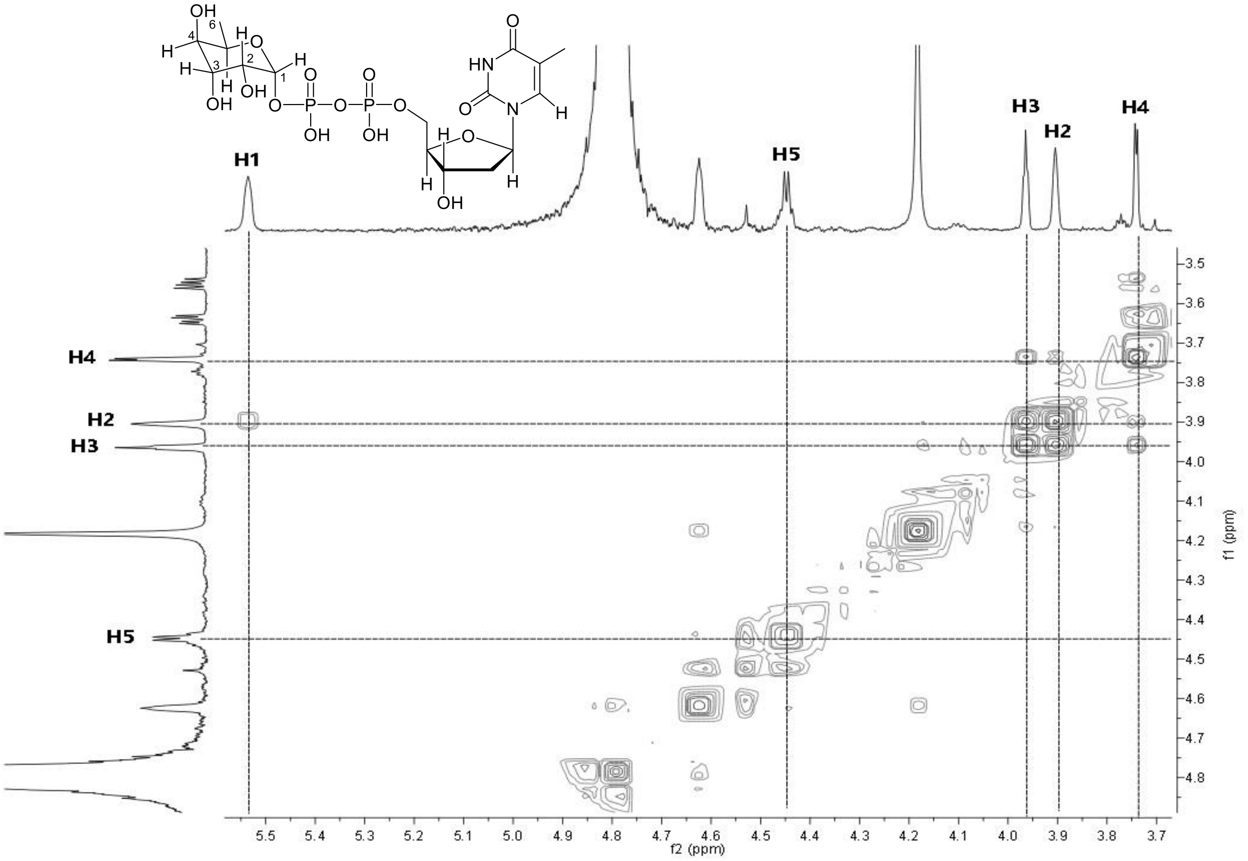

.

Figure S4. Enlargerd.^{1}H-^{1}H COSY NMR spectrum of compound **4** in D_2O.

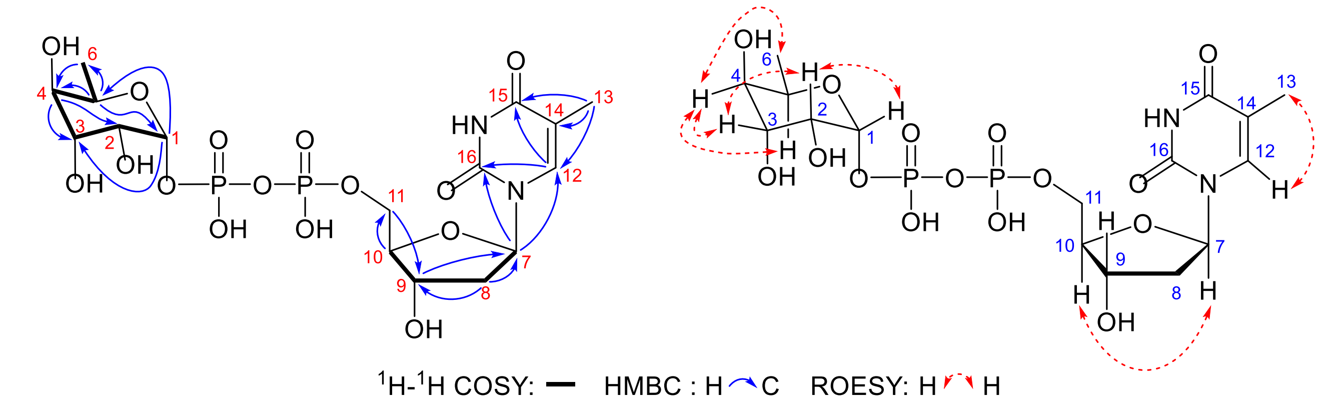

Figure S5. Key ^1H-^1H COSY, HMBC and ROESY correlations for compound **4**.

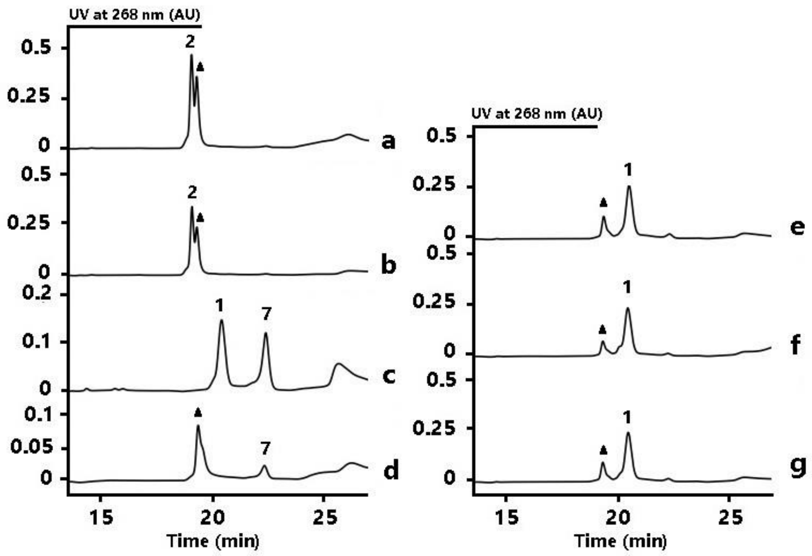

Figure S6. HPLC analysis of the products by enzymes. **a** Fcd reaction. **b** RubS3 reaction with substrate **2**. **c** FdtA reaction. **d** RubS3 reaction with substrate **7**. RubS3 homologues reaction: **e** SFQ20469. **f** WP009948706. **g** WP094006909.Triangle = NADP$^+$.

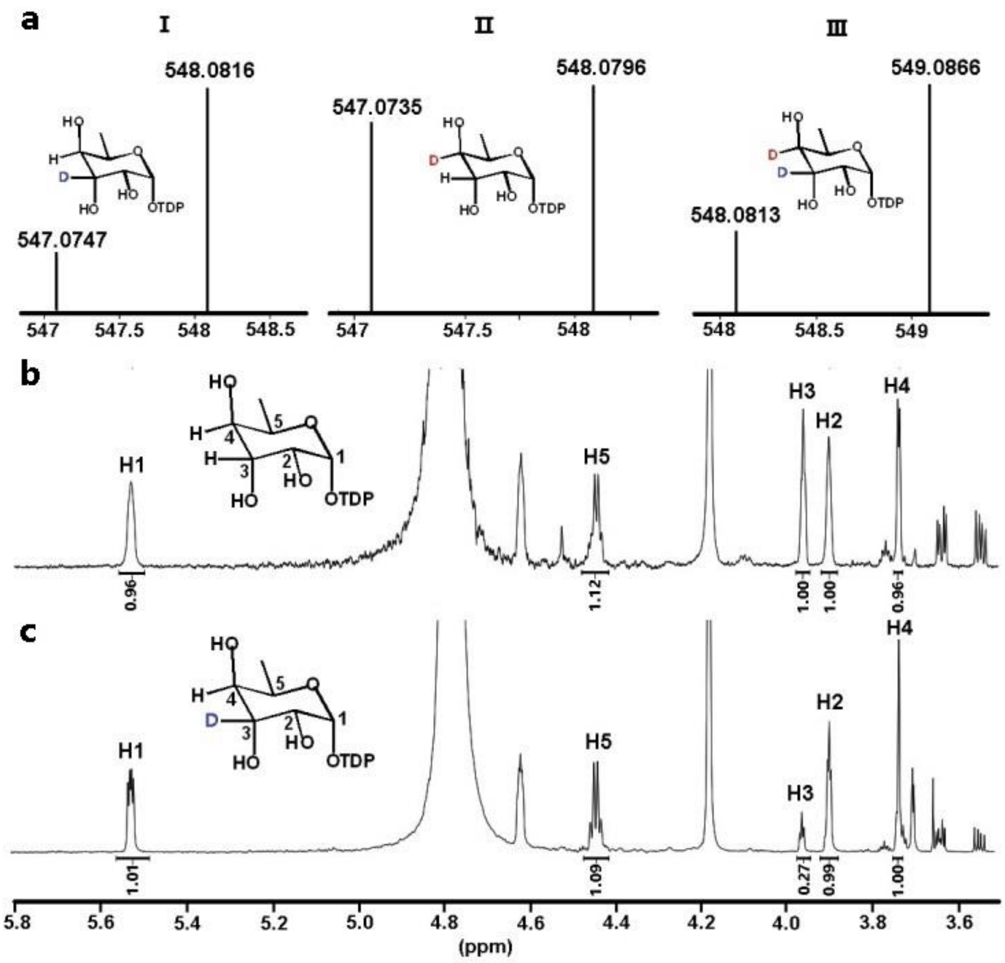

Figure S7. Deuterium exchange experiments. **a** HRESIMS analysis. I, TDP-D-antiarose formed with RubS3 in D_2O; II, TDP-D-antiarose formed with RubS3 and [4*S*-^{2}H]NADPH as cofactor; III, TDP-D-antiarose formed with RubS3 in D_2O and [4*S*-^{2}H]NADPH as cofactor. **b** ^{1}H-NMR of TDP-D-antiarose formed with RubS3 in H_2O. **c** ^{1}H-NMR of TDP-D-antiarose formed with RubS3 in D_2O.

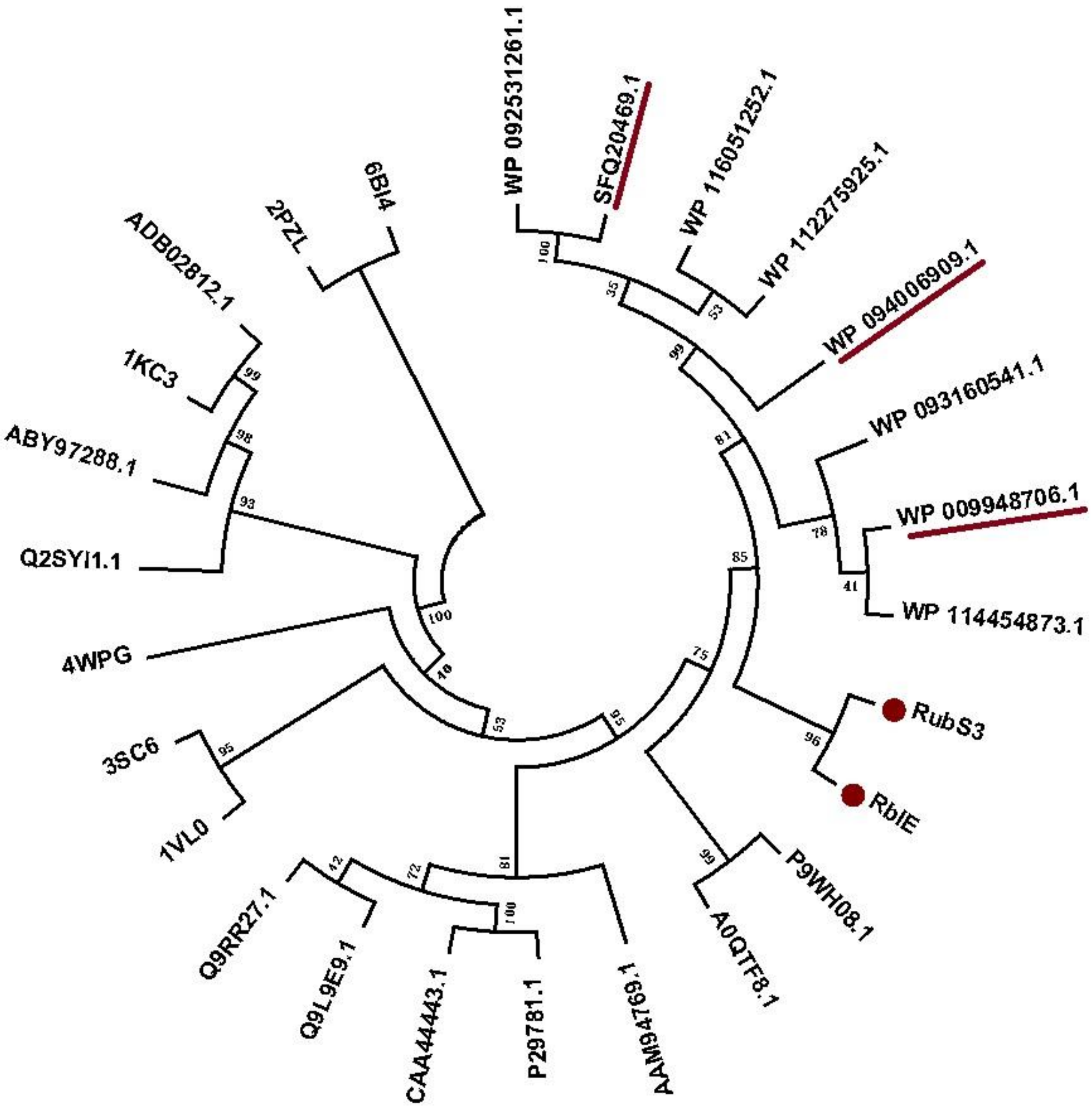

Figure S8. Phylogenetic analysis of RubS3 and its selected homologues. Sequences including two NDP-glucose-4,6-dehydratases (6BI4, RfbB from *B. anthracis*; 2PZL, WbmG from *B. bronchiseptica*) and four TDP-4-dehydrorhamnose reductases (3SC6, RfbD from *B. anthracis*; 4WPG, RmlD from *Streptococcus pyogenes*; 1KC3, RmlD from *Salmonella enterica*; 1VL0, RfbD from *Clostridium acetobutylicum*) are from Protein Data Dank (PDB). Others are selected homologues indicated by GenBank accession code.

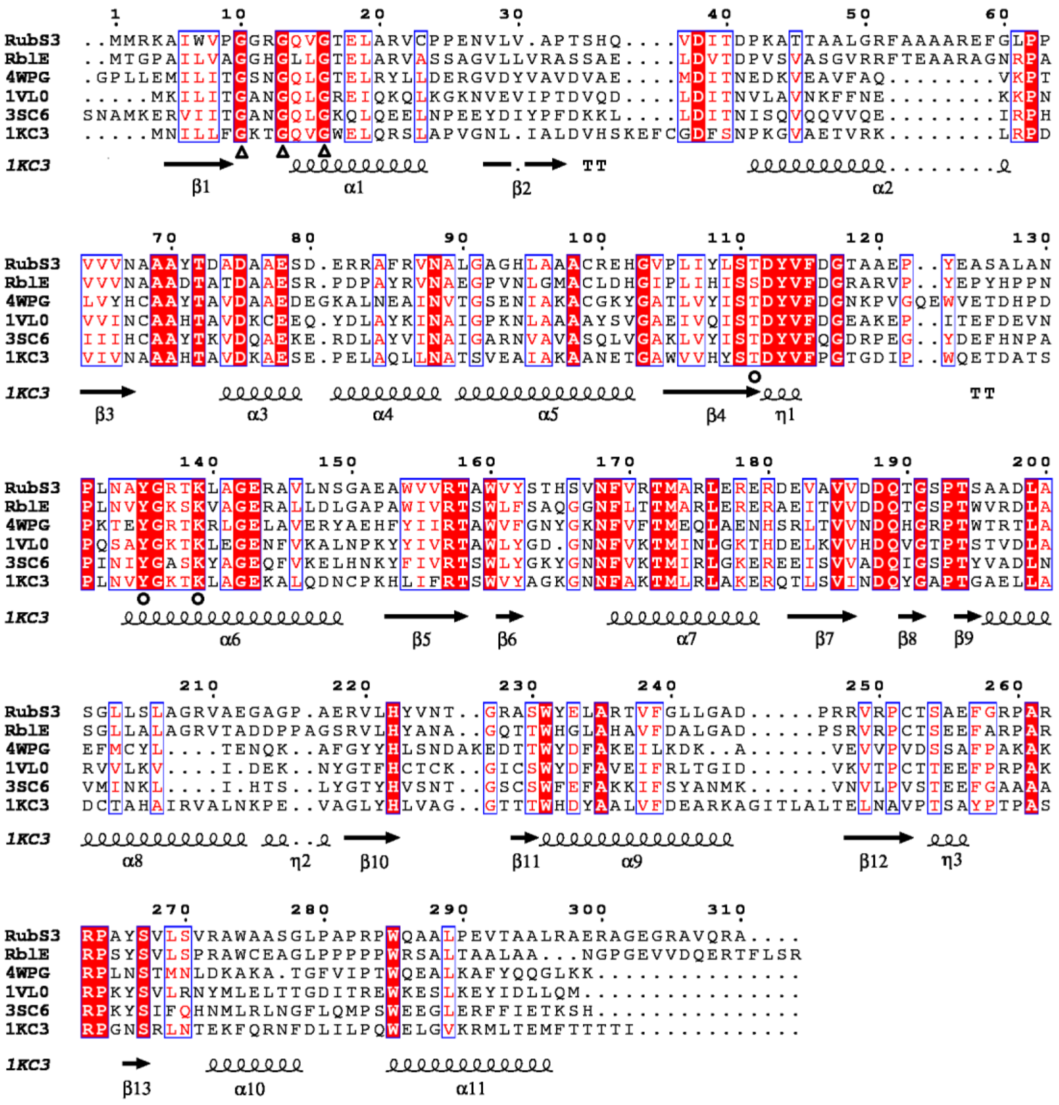

Figure S9. Alignment of the amino acid sequences for RubS3, RblE and four TDP-4-dehydrorhamnose reductases (1KC3, RmlD from *Salmonella enterica*; 4WPG, RmlD from *Streptococcus pyogenes*; 1VL0, RfbD from *Clostridium acetobutylicum*; 3SC6, RfbD from *B. anthracis*). The conserved GXXGXXG motif and catalytic TYK triad residues are indicated by triangle and circular, respectively.

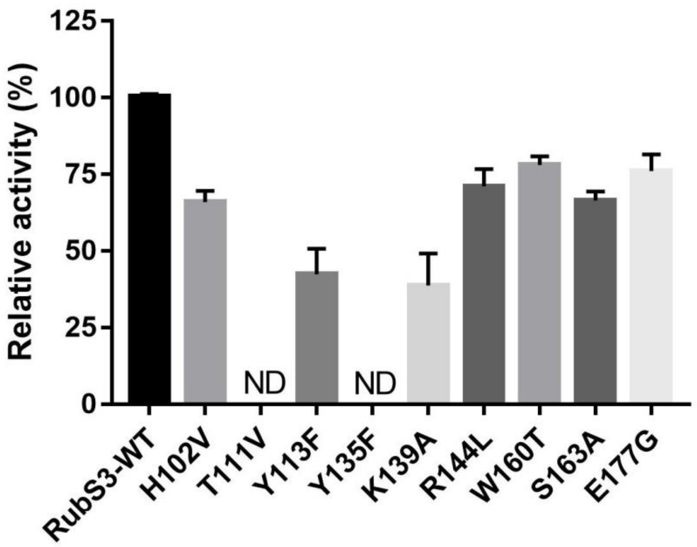

Figure S10. In vitro enzyme assays of mutants in parallel with RubS3-WT (wild type). All assays were conducted in duplicate and the relative activities were calculated from peak area ratios of product **4** between RubS3-WT and mutants. ND, the activity was not detected.

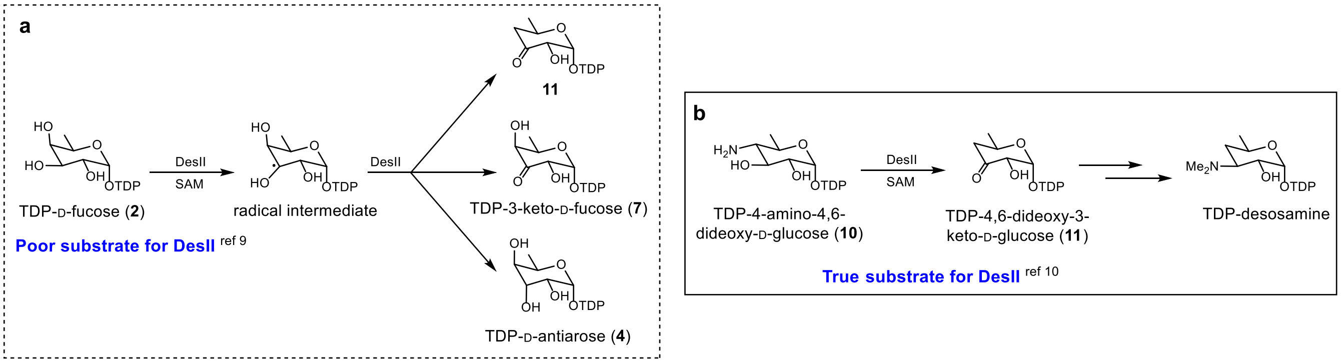

Figure S11. Radical *S*-adenosylmethionine dependent enzyme DesII-catalyzed reactions. **a** DesII can synthesize TDP-D-antiarose (**4**), TDP-3-keto-D-fucose (**7**), and TDP-4,6-dideoxy-3-keto-D-glucose (**11**) using the poor substrate TDP-D-fucose (**2**), which is not related to natural products biosynthesis. **b** DesII reaction using the true substrate **10** to generate the intermediate **11** in natural product biosynthesis.

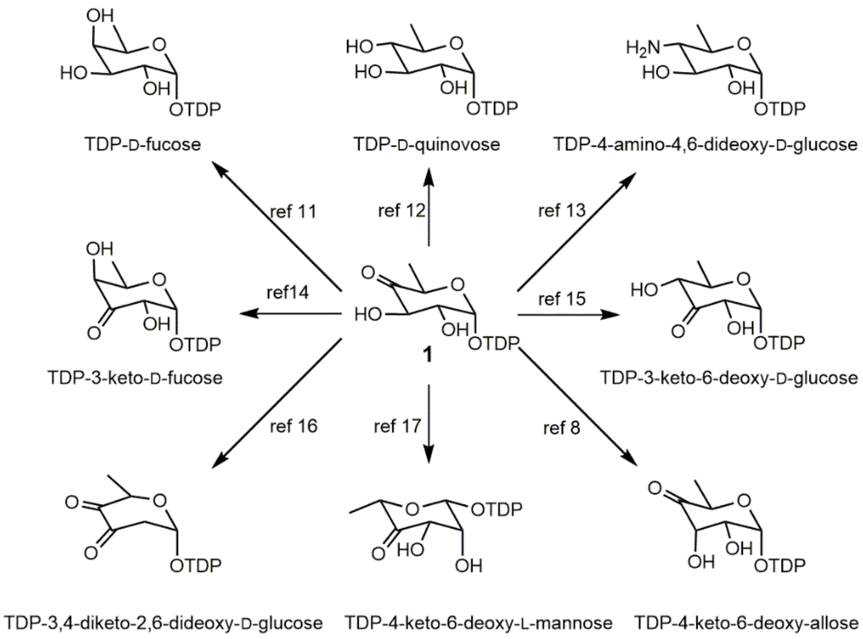

Figure S12. TDP-deoxysugars directly formed from TDP-4-keto-6-deoxy-D-glucose (**1)** by different reactions.

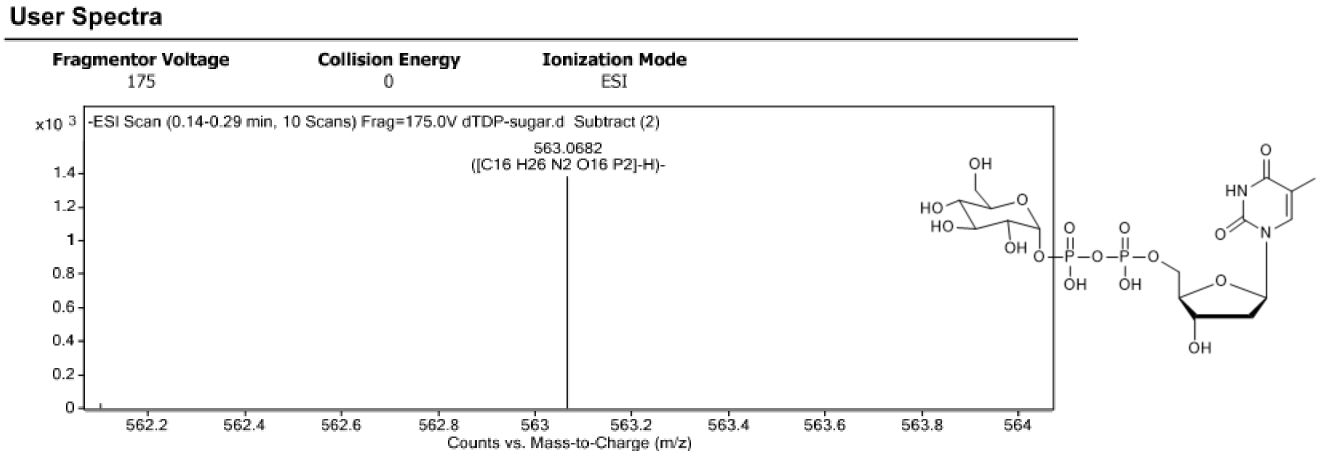

Figure S13. HRESIMS analysis of compound **3**.

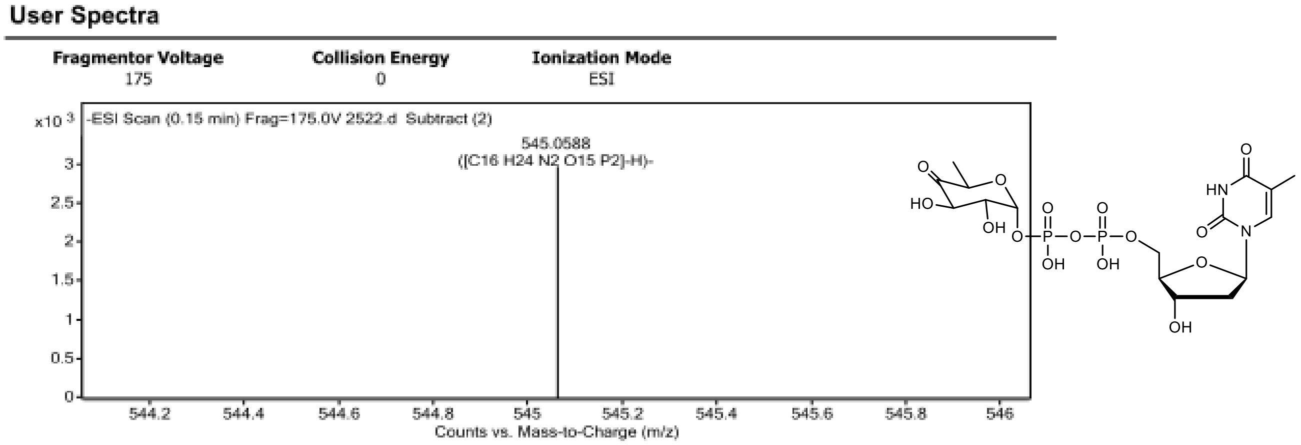

Figure S14. HRESIMS analysis of compound **1**.

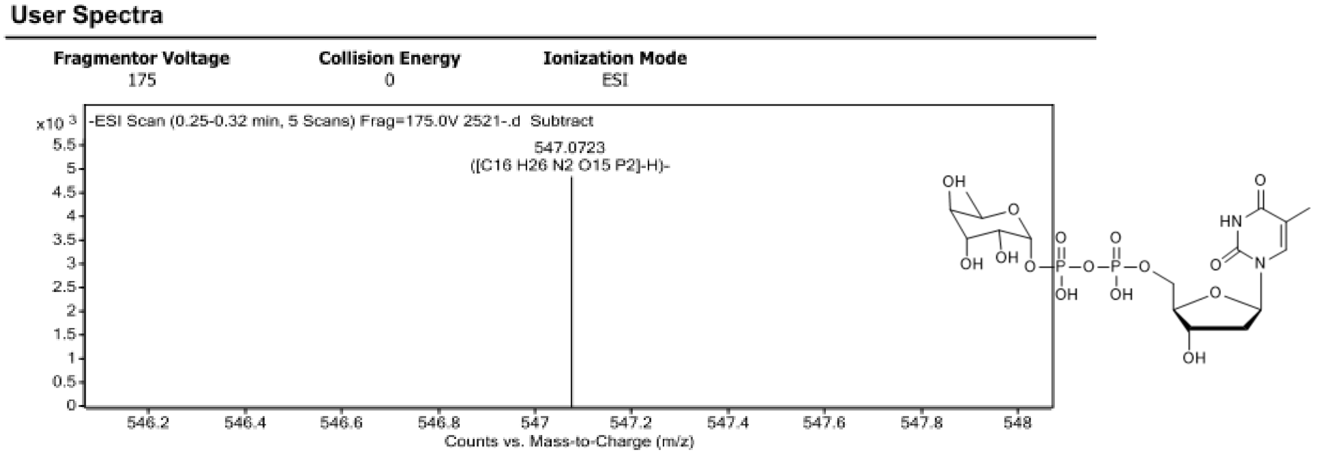

Figure S15. HRESIMS analysis of compound **4**.

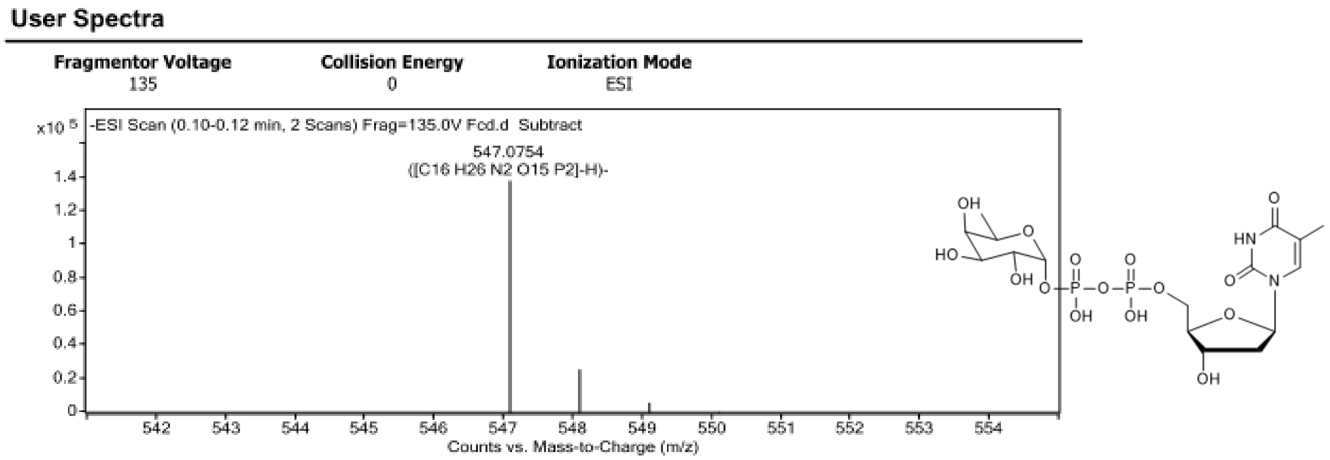

Figure S16. HRESIMS analysis of compound **2**.

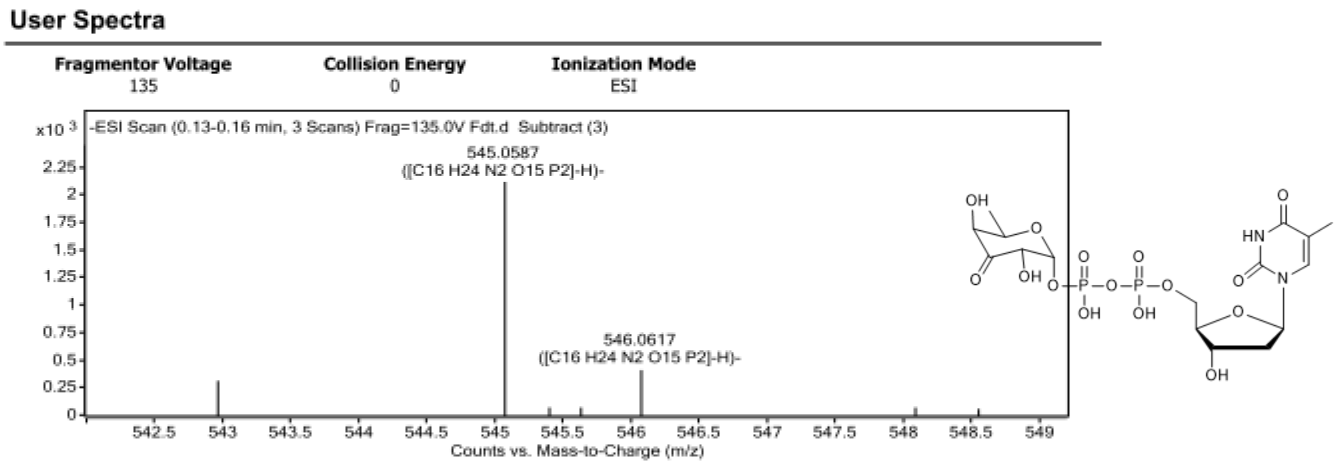

Figure S17. HRESIMS analysis of compound **7**

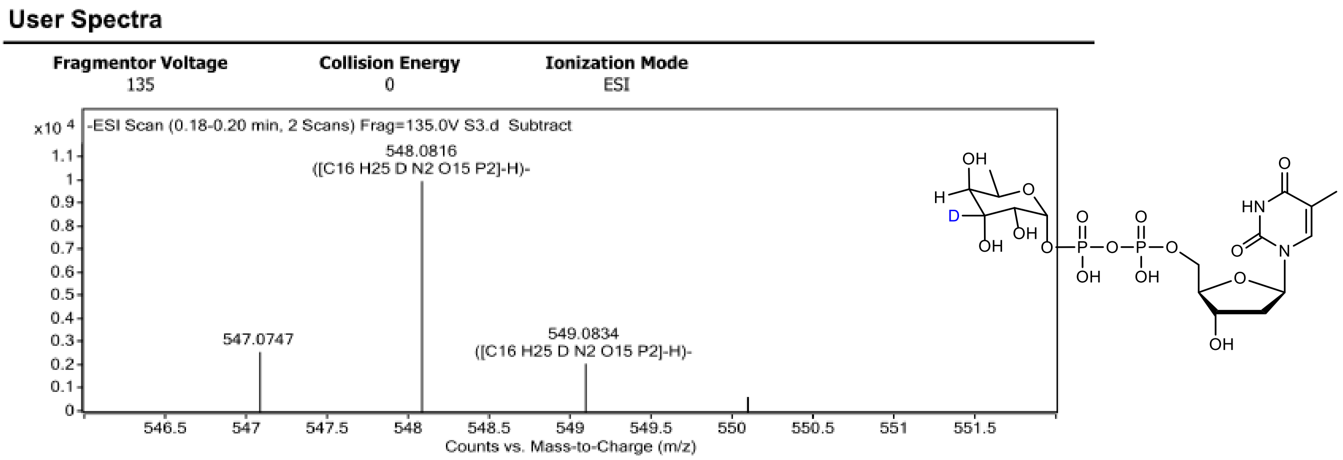

Figure S18. HRESIMS analysis of compound **4'** formed with RubS3 in D_2O

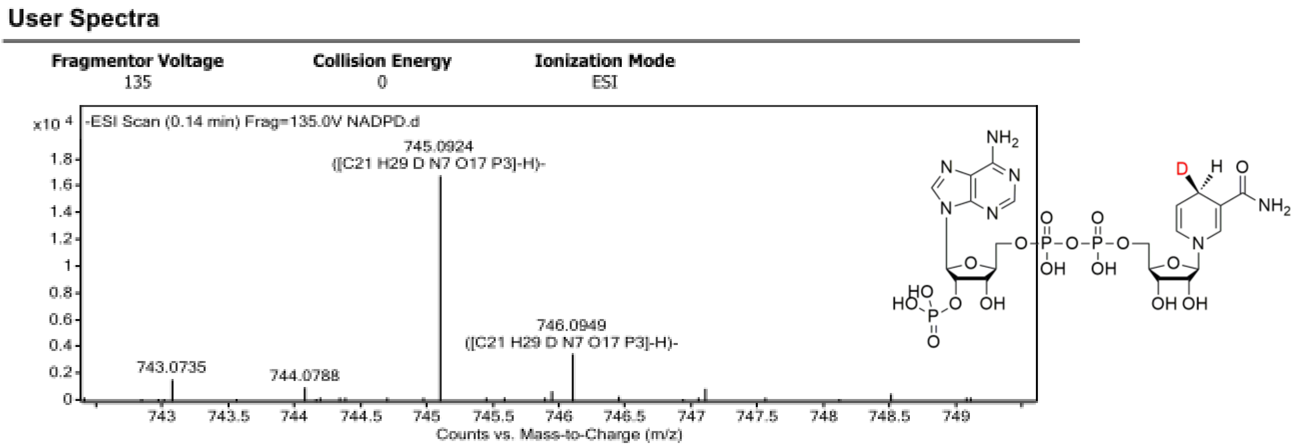

Figure S19. HRESIMS analysis of synthesized [4*S*-^{2}H] NADPH.

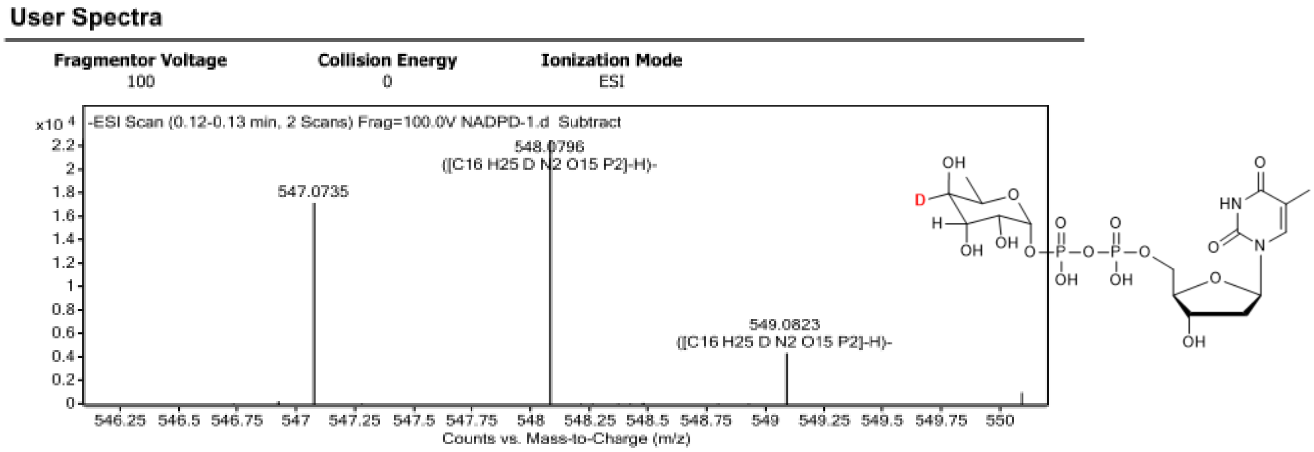

Figure S20. HRESIMS analysis of **4”** formed with RubS3 use [4*S*-^{2}H] NADPH as cofactor

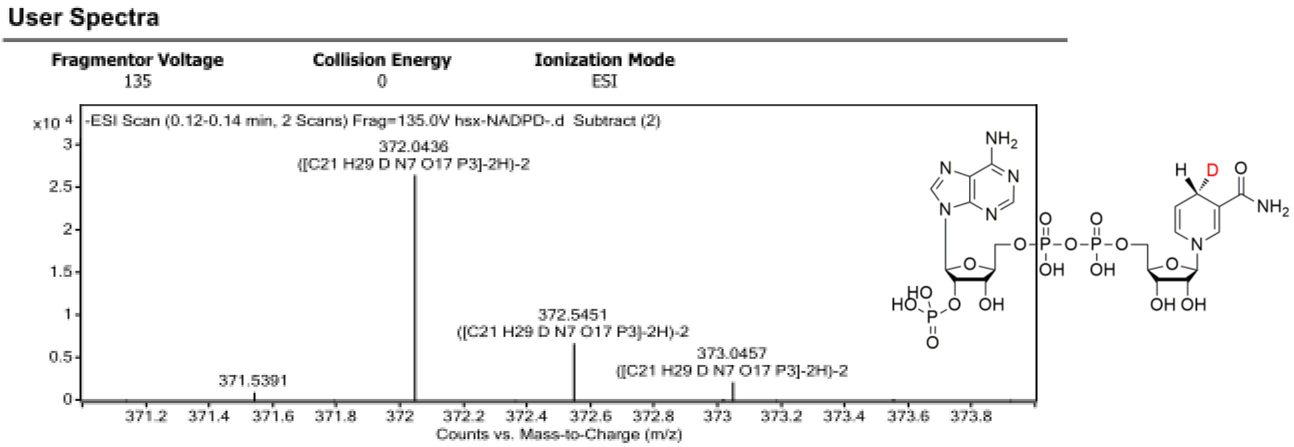

Figure S21. HRESIMS analysis of synthesized [4*R*-^{2}H] NADPH.

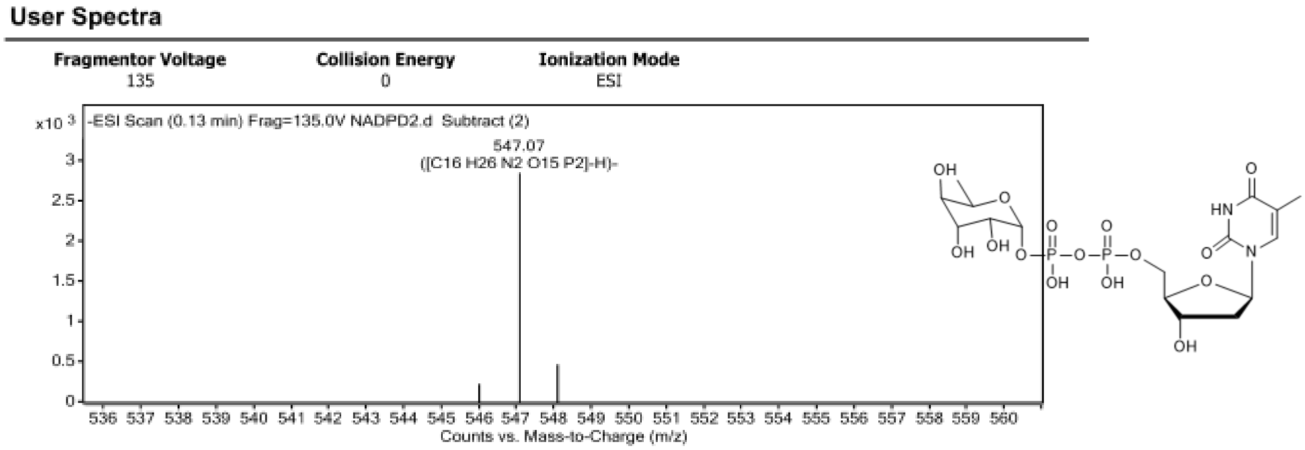

Figure S22. HRESIMS analysis of compound **4** formed with RubS3 use [4*R*-^{2}H] NADPH as cofactor.

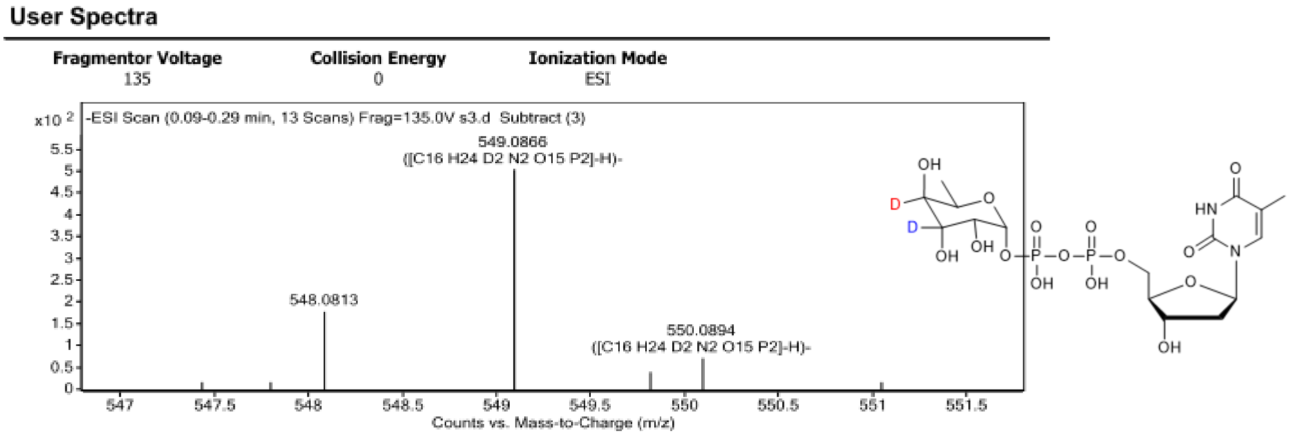

Figure S23. HRESIMS analysis of compound **4'''** formed with RubS3 in D_2O and use [4*S*-^{2}H]NADPH as cofactor.

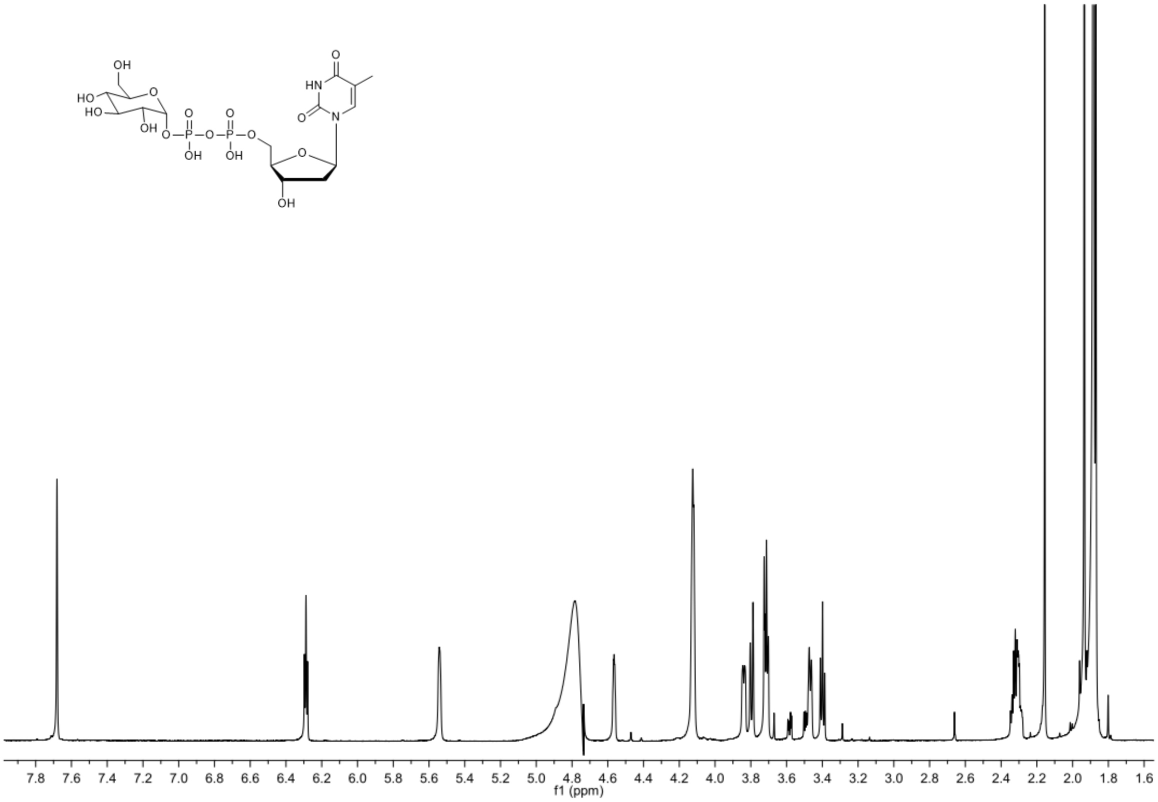

Figure S24. ^{1}H NMR spectrum of compound **3** in D_2O.

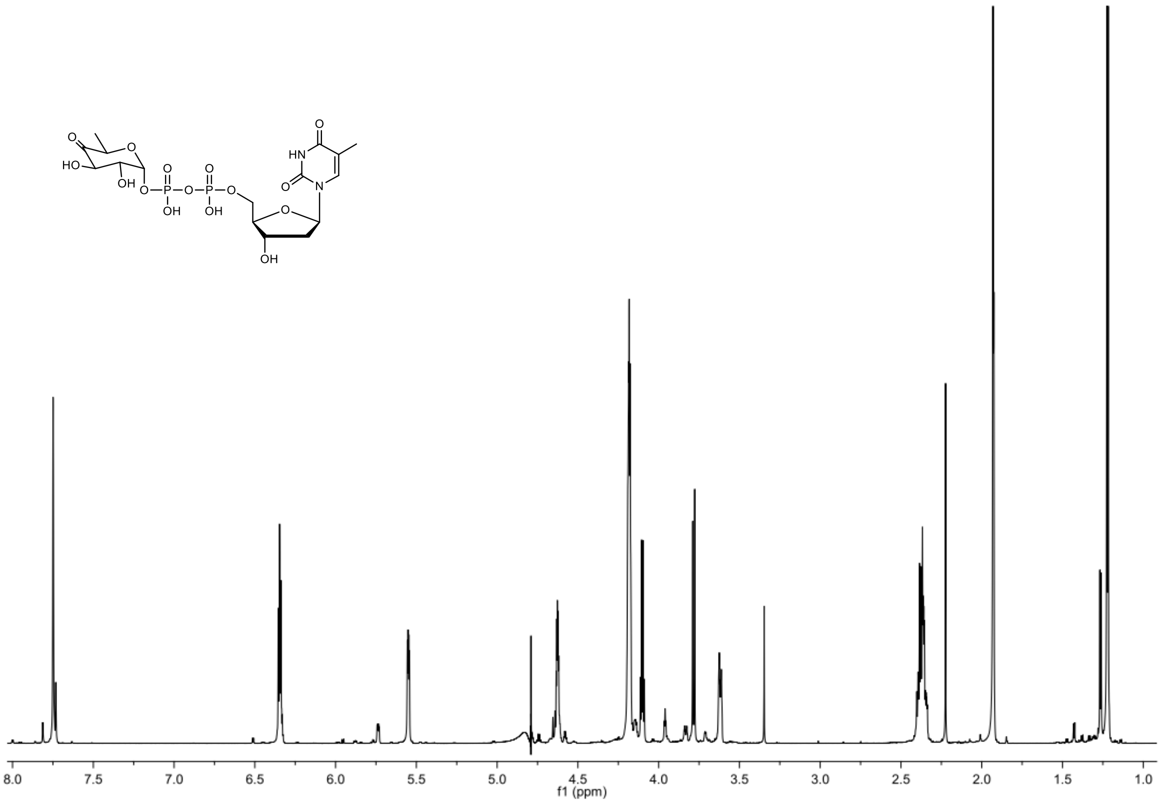

Figure S25. ^{1}H NMR spectrum of compound **1** in D_2O

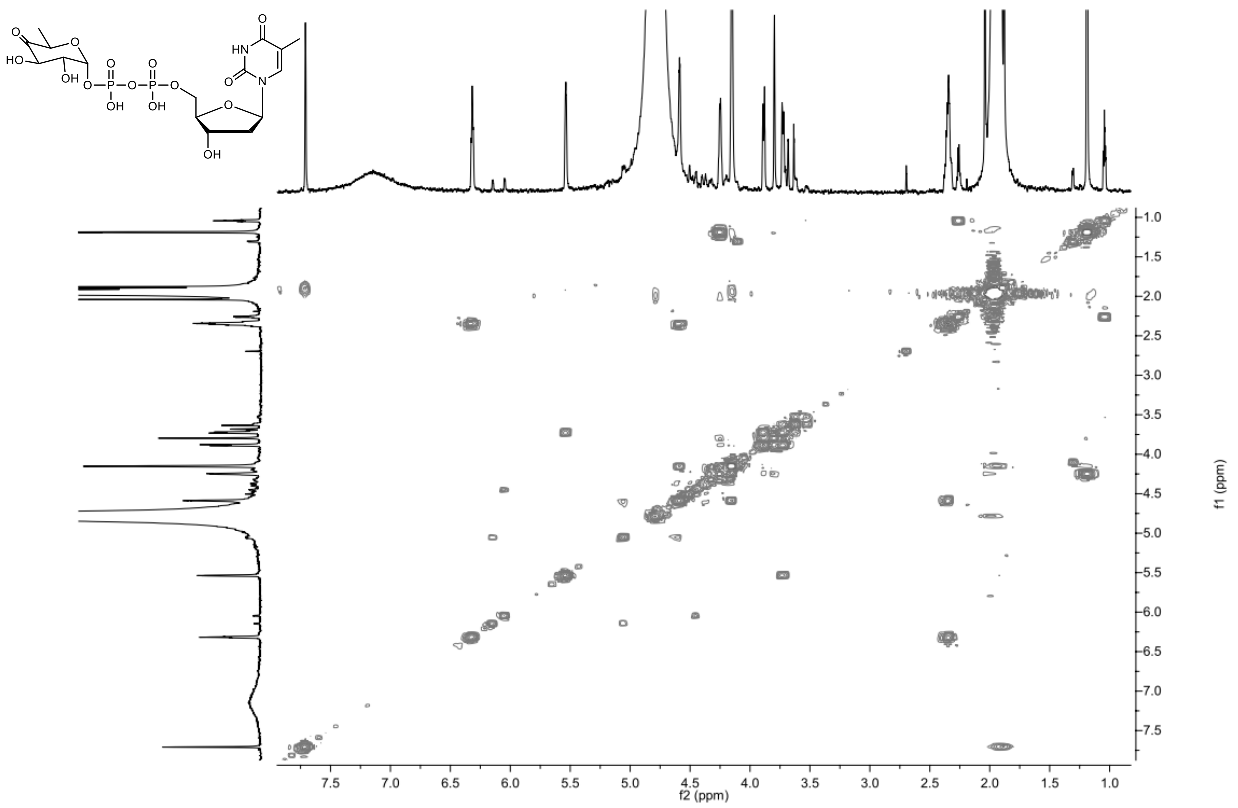

Figure S26. ^{1}H-^{1}H COSY NMR spectrum of compound **1** in D_2O.

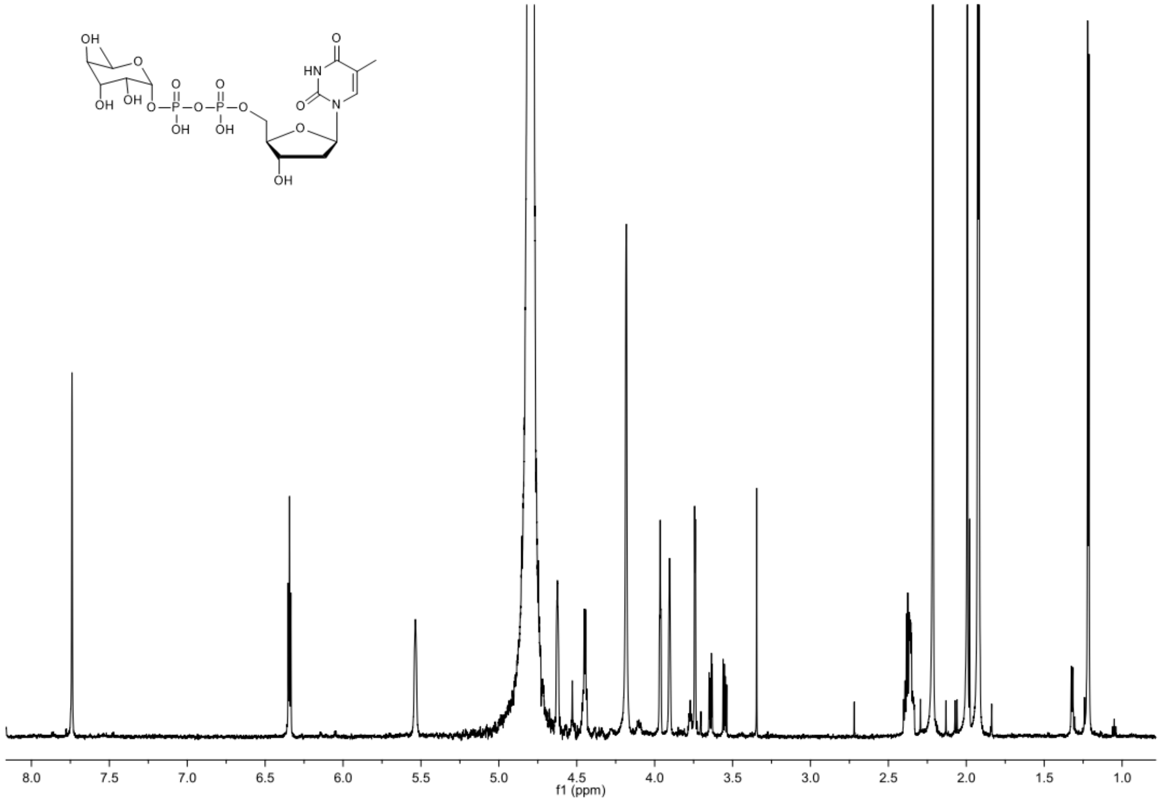

Figure S27. ^{1}H NMR spectrum of compound **4** in D_2O.

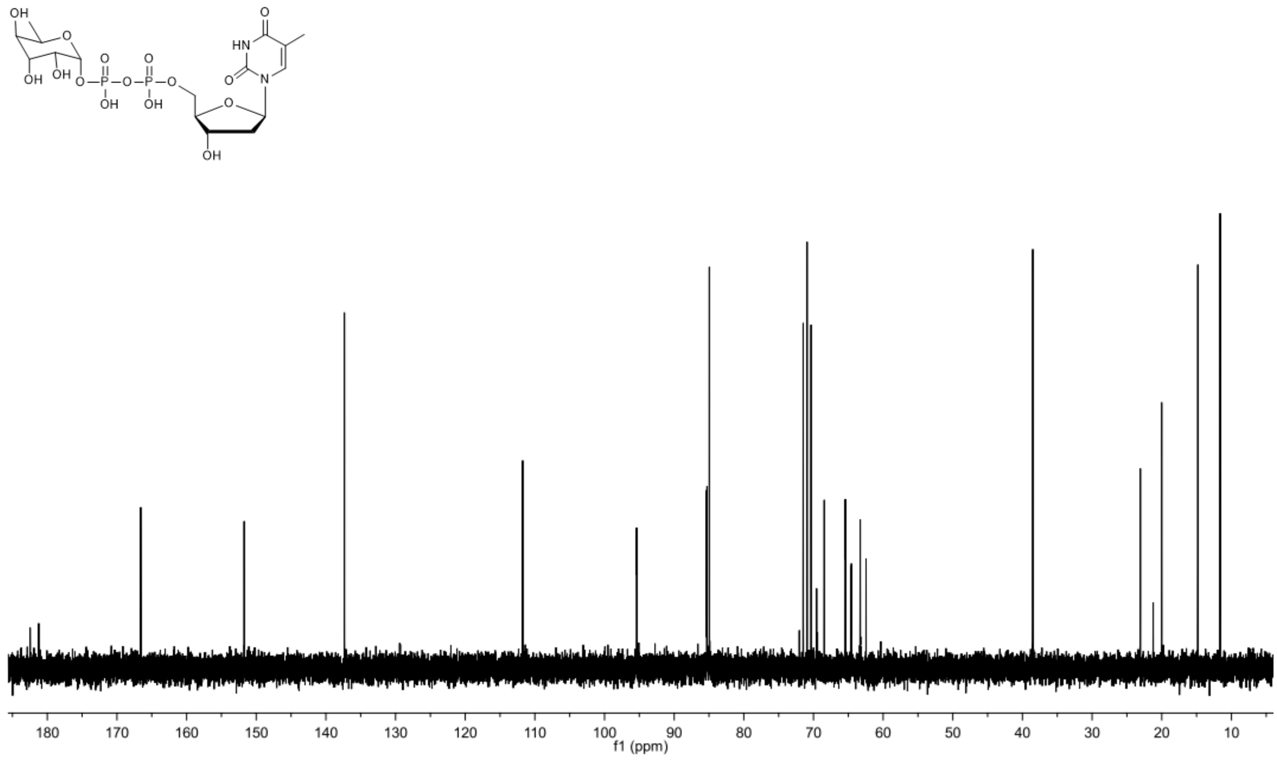

Figure S28. ^{13}C NMR spectrum of compound **4** in D_2O.

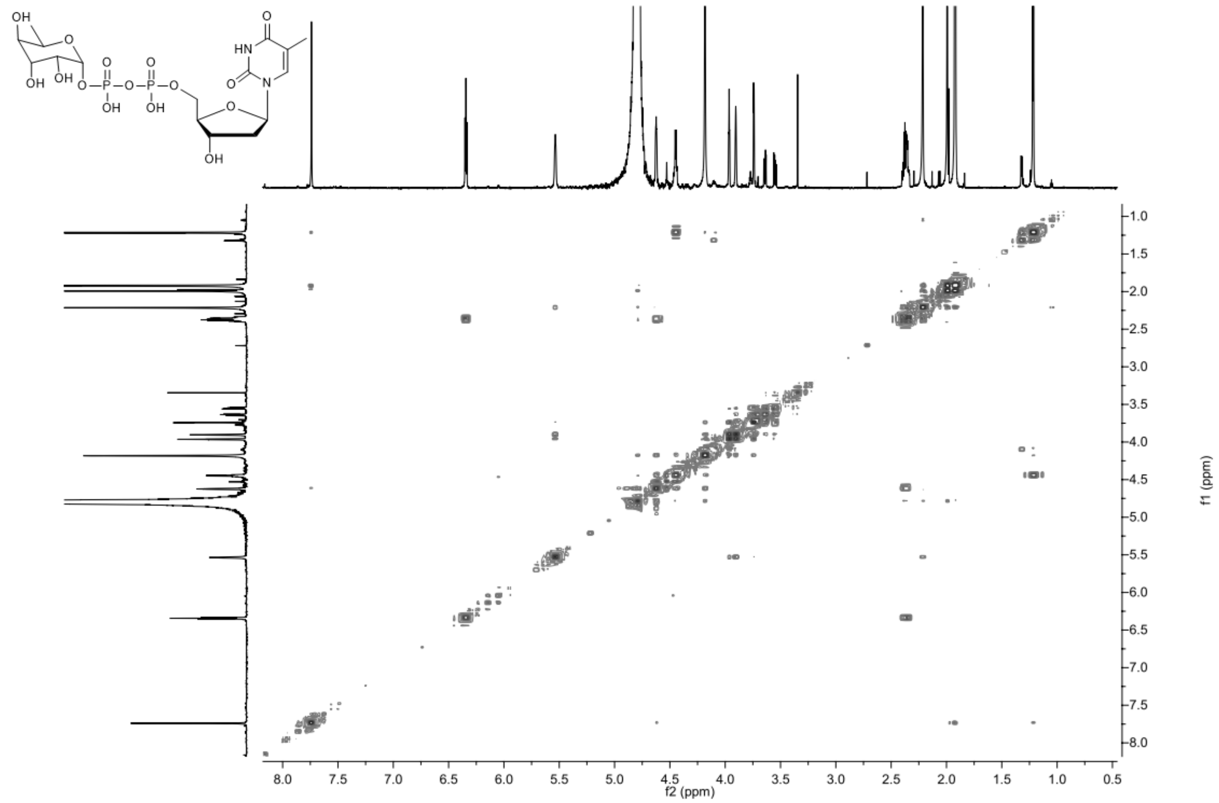

Figure S29. ^1H-^1H COSY NMR spectrum of compound **4** in D_2O.

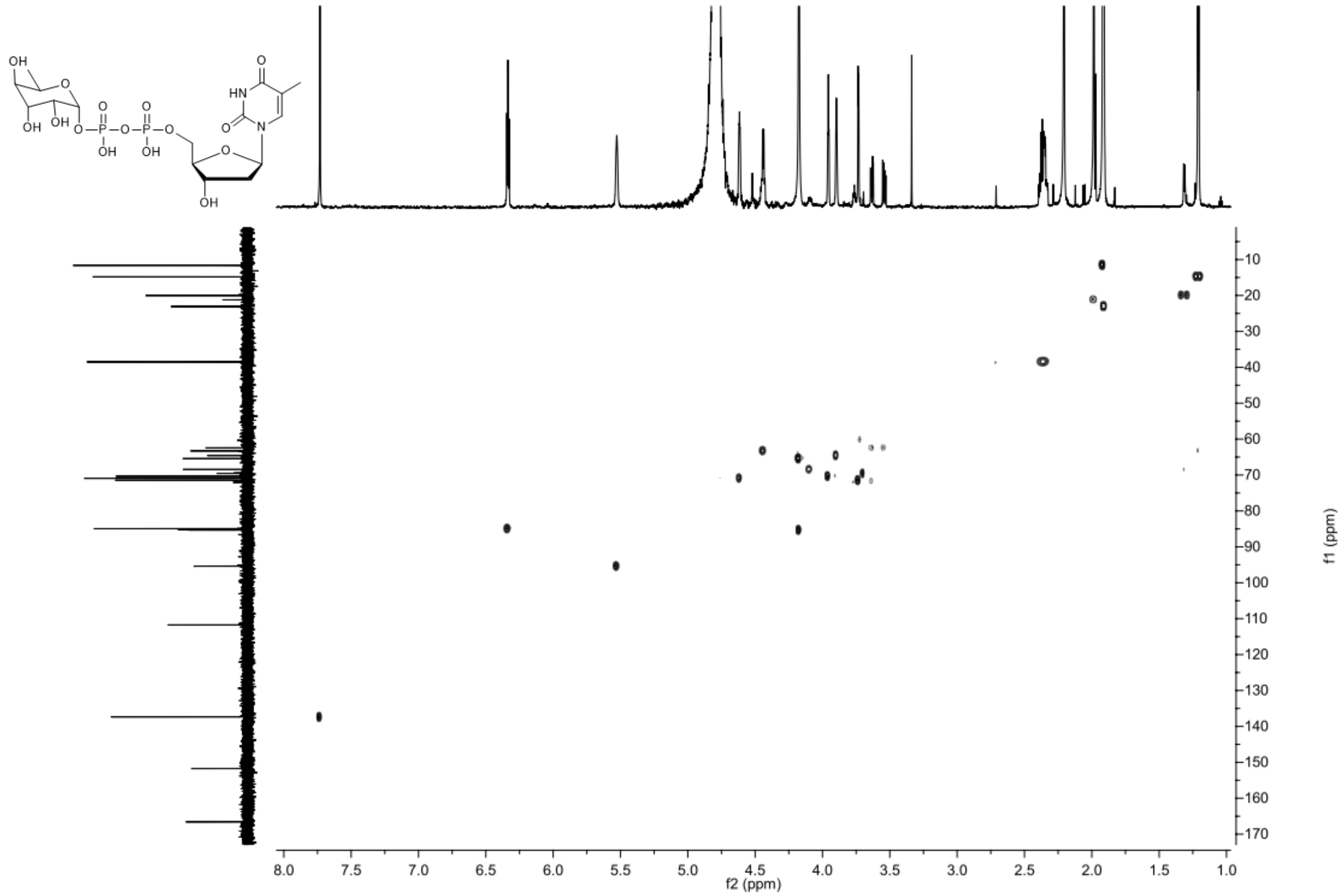

Figure S30. HSQC NMR spectrum of compound **4** in D_2O.

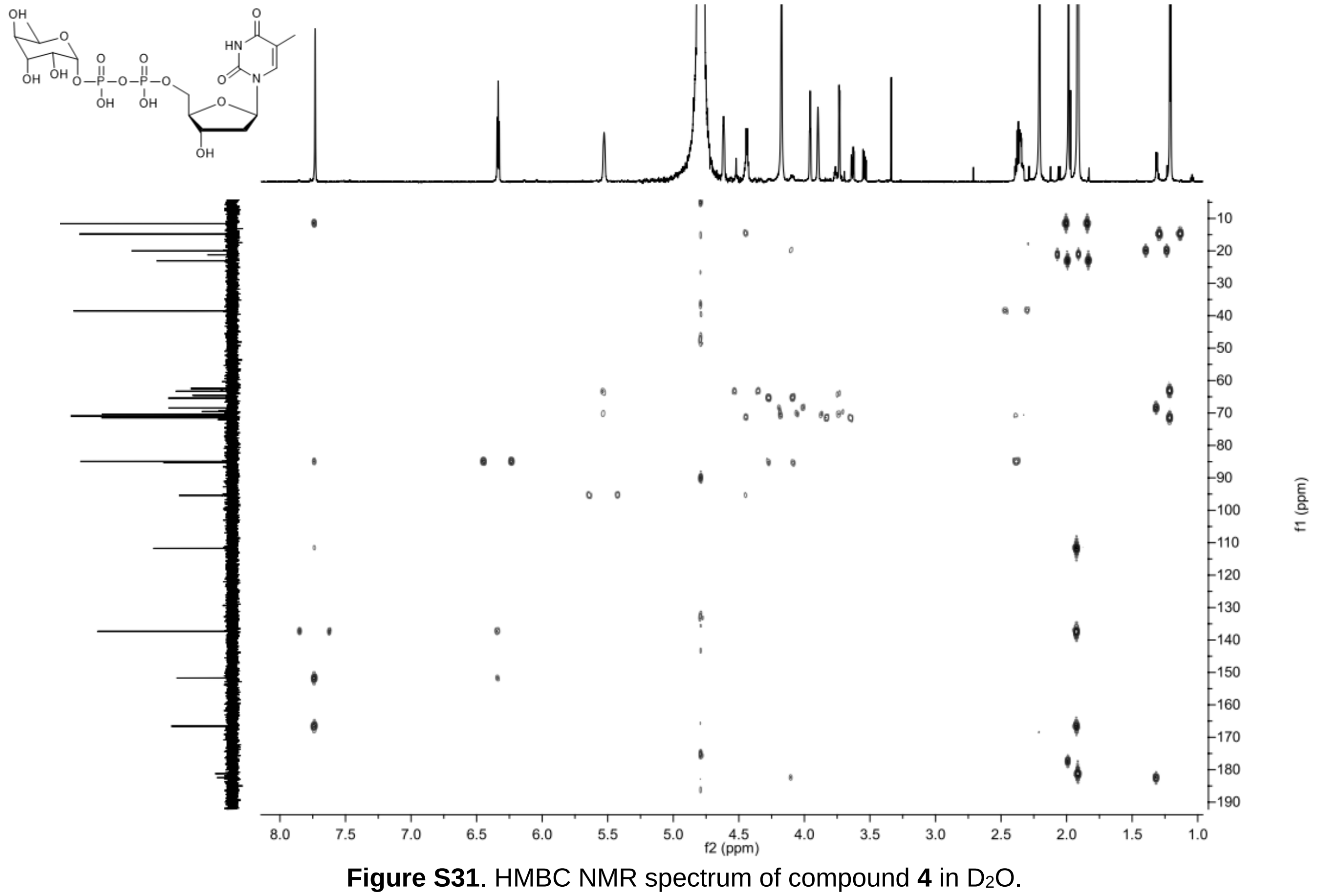

Figure S31. HMBC NMR spectrum of compound **4** in D_2O.

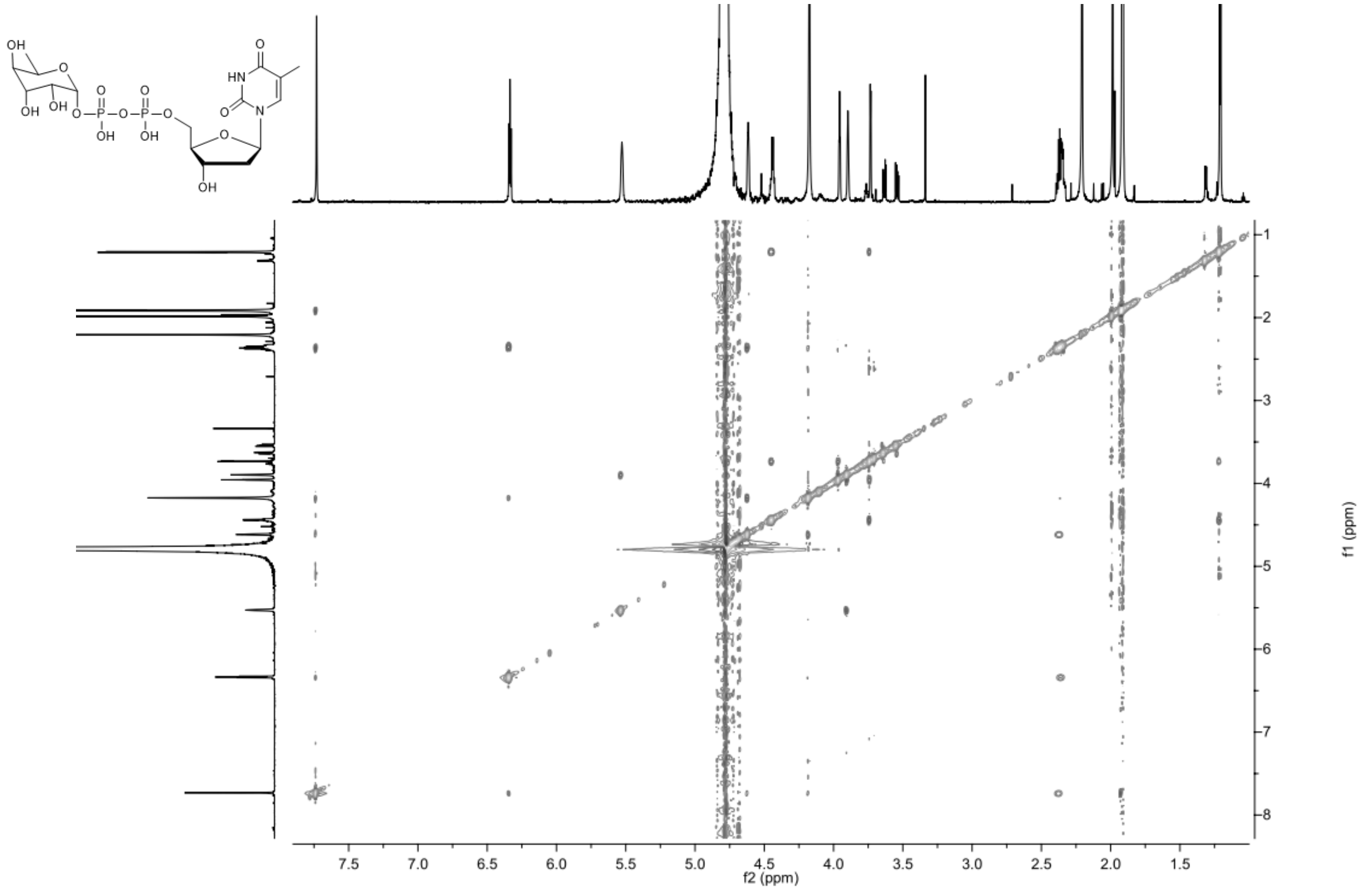

Figure S32. ROESY NMR spectrum of compound **4** in D_2O.

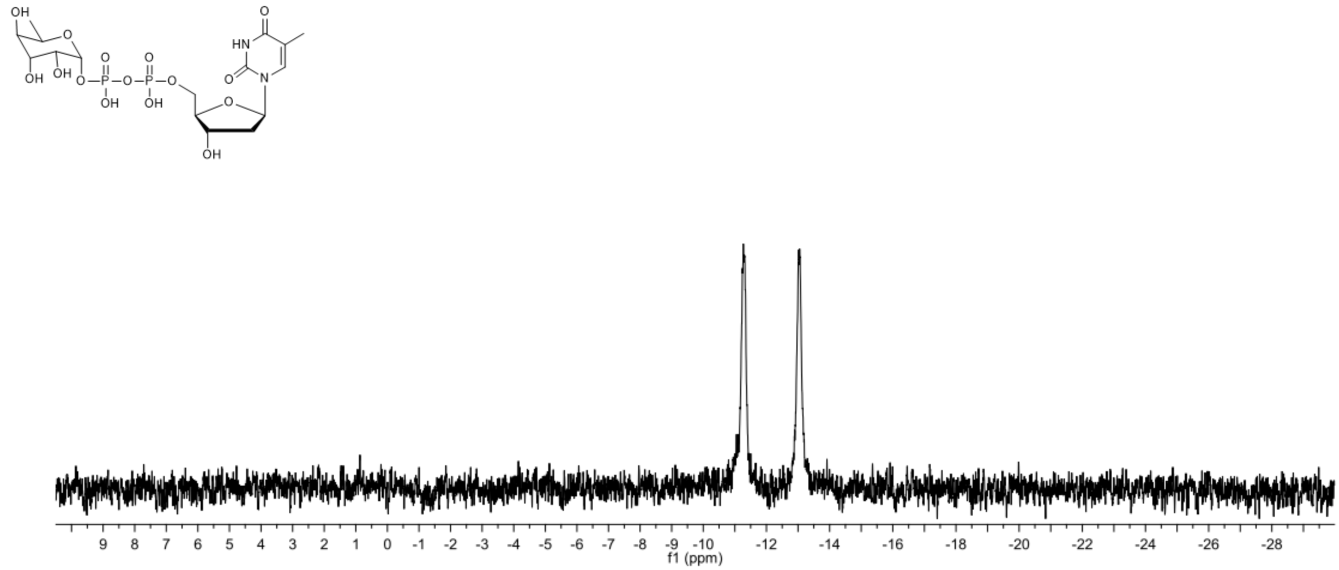

Figure S33. ^{31}P NMR spectrum of compound **4** in D_2O.

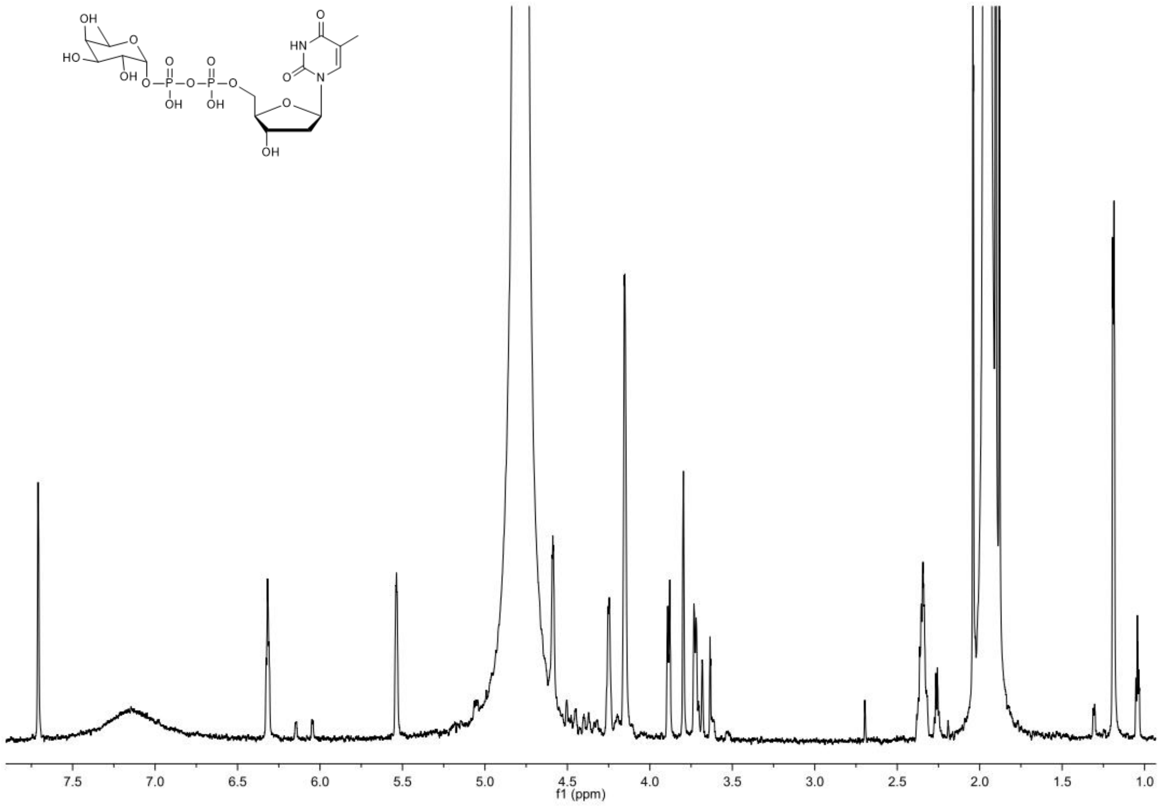

Figure S34. ^{1}H NMR spectrum of compound **2** in D_2O.

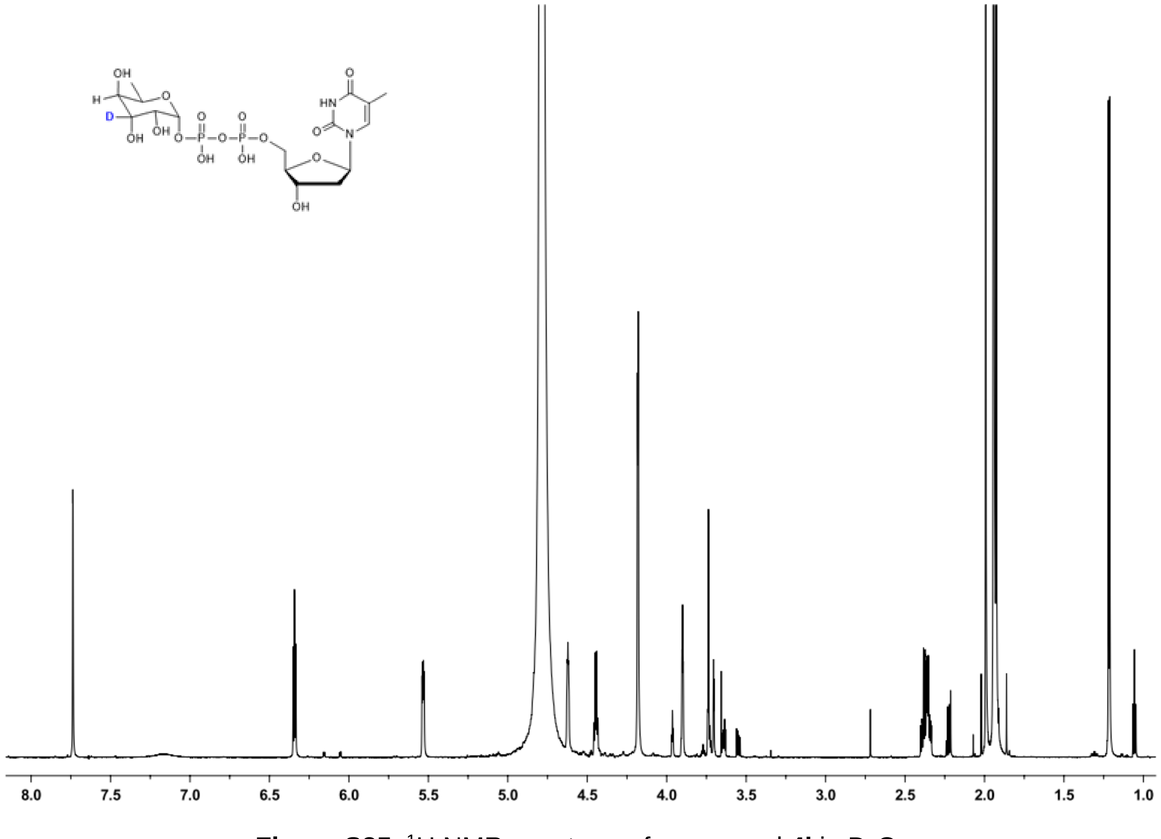

Figure S35. ^{1}H NMR spectrum of compound **4’** in D_2O.

References

1. Y. Yan, J. Yang, Z. Yu, M. Yu, Y.-T. Ma, L. Wang, C. Su, J.-Y., Luo, G. P. Horsman and S.-X. Huang, *Nat. Commun.,* 2016, **7**, 13083.
2. V. V. Pollock and M. J. Barber, *Biochemistry,* 2001, **40**, 1430-1440.
3. J. Felsenstein, *Evolution,* 1985, **39**, 783-791.
4. S. Kumar, K. Tamura and M. Nei, *Brief. Bioinform*., 2004, **5**, 150-163.
5. F. Madeira, Y. M. Park, J. Lee, N. Buso, T. Gur, N. Madhusoodanan, P. Basutkar, A. R. N. Tivey, S. C. Potter, R. D. Finn and R. Lopez, *Nucl. Acids Res.,* 2019*,* **47**, 636-641.
6. X. Robert and P. Gouet, *Nucl. Acids Res*., 2014, **42**, 320-324.
7. L. Elling, C. Rupprath, N. Günther, U. Römer, S. Verseck, P. Weingarten, G. Drager, A. Kirschning and W. Piepersberg, *ChemBioChem,* 2005, **6**, 1423-1430.
8. T. T. T. Thuy, K. Liou, T.-J. Oh, D.-H. Kim, D.-H. Nam, J.-C. Yoo and J.-K. Sohng, *Glycobiology,* 2007, **17**, 119-126.
9. Y. Ko, M. W. Ruszczycky, S.-H. Choi and H.-W. Liu, *Angew. Chem. Int. Ed.,* 2015, **54**, 860-863.
10. P.-H. Szu, M. W. Ruszczycky, S.-H. Choi, F. Yan and H.-W. Liu, *J. Am. Chem. Soc.,* 2009, **131**, 14030-14042.
11. Y. Yoshida, Y. Nakano, T. Nezu, Y. Yamashita and T. Koga, *J. Biol. Chem.,* 1999, **274**, 16933-16939.
12. S. A. Borisova, L. Zhao, D. H. Sherman and H.-W. Liu, *Org. Lett.,* 1999, **1**, 133-136.
13. Y.-S. Chung, D.-H. Kim, W.-M. Seo, H.-C. Lee, K. Liou, T.-J Oh and J.-K Sohng, *Carbohydr. Res.,* 2007, **34**, 1412-1418.
14. A. Pfoestl, A. Hofinger, P. Kosma and P. Messner, *J. Biol. Chem.,* 2003, **278**, 26410-26417.
15. C. E. Melancon, L. Hong, J. A. White, Y.-N. Liu and H.-W. Liu, *Biochemistry,* 2007, **46**, 577-590.
16. H.-W. Chen, G. Agnihotri, Z. Guo, N. L. S Que, X.-H. Chen and H.-W. Liu, *J. Am. Chem. Soc.,* 1999, **121**, 8124-8125.
17. M. Graninger, B. Nidetzky, D. E. Heinrichs, C. Whitfield and P. Messner, *J. Biol. Chem*., 1999, **274**, 25069-25077.
